# Insights into CO_2_ Adsorption Motifs in Functionalized Mesoporous Silica Using Solid-State NMR

**DOI:** 10.1021/acsomega.6c04679

**Published:** 2026-08-12

**Authors:** Mohammed Jasil, Athulya Nadol, Hyeseung Jeong, Brijith Thomas

**Affiliations:** † Department of Chemistry, 167632 New York University, New York, New York 10003, United States; ‡ Science Division, New York University Abu Dhabi (NYUAD), Abu Dhabi 129188, United Arab Emirates

## Abstract

The immobilization of amine active sites onto porous supports, such as polyethylenimine (PEI) impregnated mesoporous silica, is well explored for carbon dioxide adsorption. However, a qualitative investigation of the molecular-level interactions among PEI, water molecules, and CO_2_ within the pore walls of mesoporous silica remains challenging. Probing such interactions requires spectroscopic methods that can access the local environment of the PEI/silica composite. A systematic investigation is needed to elucidate the atomistic picture of physically impregnated PEI within silica pores, particularly in relation to CO_2_ adsorption processes. Based on existing literature and feasible scenarios, we formed three distinct cases for the physical impregnation of PEI into the pores of silica. We synthesized a model system comprising porous silica impregnated with PEI at various weight percentages. We performed solid-state magic angle spinning (MAS) nuclear magnetic resonance (NMR) spectroscopy experiments on a series of PEI-impregnated porous silica materials. The observed peak shifts and line shape changes in the ^1^H and ^13^C MAS NMR spectra, together with the complementary spectroscopic data, indicate that PEI forms a conformal layer along the silica pore walls at lower loadings and agglomerates within the pores at higher loadings. Moreover, PEI agglomeration becomes pronounced at PEI loadings above 40 wt %. A custom-built CO_2_ adsorption setup was used to perform CO_2_ adsorption on PEI/silica composites packed within solid-state NMR rotors. Complementary quantum mechanical calculations support the experimental observations and indicate the formation of carbamic acid species, characterized by a ^13^C resonance at 164 ppm. These molecular-level insights into the interactions within the PEI/silica CO_2_ system provide a framework for the design of high-performance amine-functionalized adsorbents for carbon capture applications.

## Graphical abstract

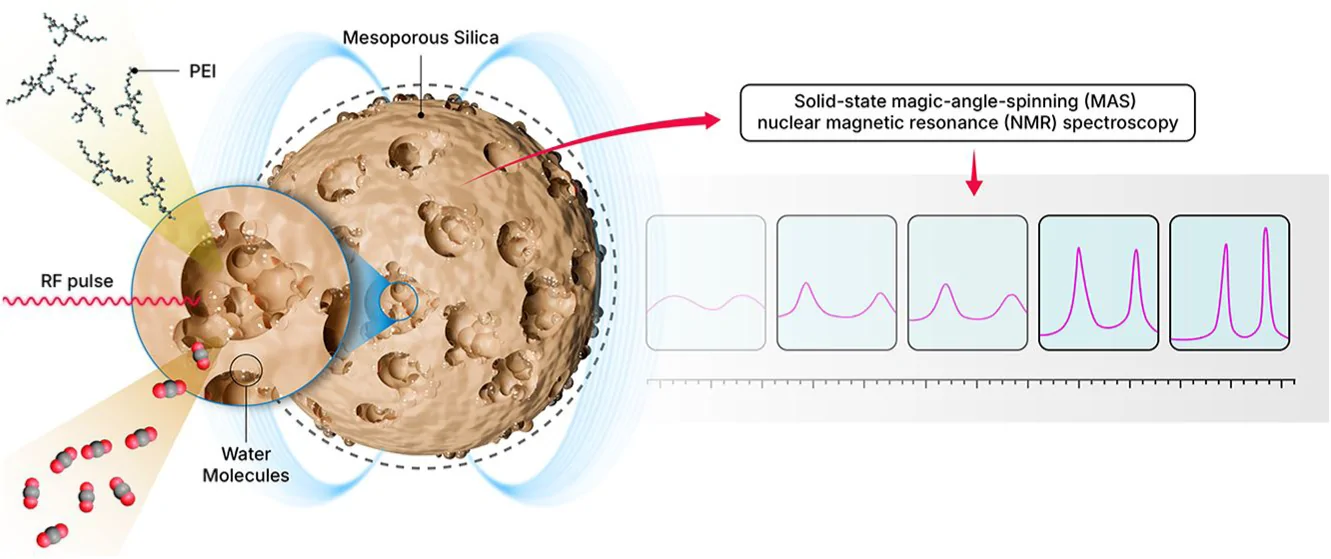

## Introduction

The capture and conversion of carbon dioxide (CO_2_) has recently emerged as a critical area of research due to its dual potential to address two key issues: the increasing levels of CO_2_ in the atmosphere and the global energy demand.^1–5^ The concentration of CO_2_ in the atmosphere has continued to rise, reaching 431.44 ppm in June 2026.^6–9^ Diverse materials have been studied recently for capturing CO_2_, including covalent organic frameworks (COFs),^10,11^ metal–organic frameworks (MOFs),^12,13^ and porous silica-based materials like (Santa Barbara Amorphous) SBA-15,^14–16^ (Mobil Composition of Matter) MCM-41,^17^ fibrous nanosilica,^18^ as well as zeolites.^19,20^ Impregnation of amine-rich polymers into porous materials is a method to functionalize the silica with active centers for CO_2_ adsorption.^21–29^

The stabilization and dynamics of confined PEI within silica pores are critical to determining the material’s CO_2_ adsorption properties. The interface between mesoporous silica and polyethylenimine (PEI) is characterized by hydrogen bond interactions or van der Waals forces between amine groups and silanol groups.,^30–34^ It is of particular interest to study the noncovalent interactions within the pore environment, which comprises amine functionalities, silica, water, and CO_2_. Previous studies have employed infrared spectroscopy (IR), small-angle X-ray scattering (SAXS), small-angle neutron scattering (SANS), and X-ray photoelectron spectroscopy (XPS) to probe the interactions between PEI and silica.^30,35,36^ However, these techniques lack the atomistic resolution required to characterize the noncovalent and covalent interactions present at the interface. In well-defined mesoporous silica materials, the pore walls are coated with a conformal layer of PEI at lower loadings, and as the loading increases, PEI forms plugs in the pore channels.^35,37,38^ At very high PEI loadings, it agglomerates on the surface. However, molecular-level interactions between PEI and the silica gel pore walls, investigated using techniques such as solid-state NMR spectroscopy, can provide detailed insights into the nature and dynamics of these interactions.

In this study, we combine spectroscopy with theoretical modeling to obtain atomic-level insight into the interaction between PEI and silica mesopores. Silica gel was selected as the silica precursor, considering the industrial scalability of CO_2_ adsorbents owing to its low cost and ready availability. A gap remains in our understanding of the noncovalent interactions of PEI within the pores of silica gel, the influence of different PEI loadings on various aqueous environments in these pores, and the formation and dynamics of CO_2_ adsorption species at different PEI loadings. In this study, we use PEI-impregnated silica gel incorporating varying weight percentages of PEI to conduct a systematic analysis. We utilize powder X-ray diffraction (PXRD), N_2_ Brunauer–Emmett–Teller (BET) adsorption isotherm measurements, thermogravimetric analysis (TGA), scanning electron microscopy (SEM), infrared spectroscopy, and solution-state nuclear magnetic resonance (NMR) spectroscopy to characterize the synthesized composites. Further elucidation of the atomistic interactions among PEI, water molecules, silica pore walls, and CO_2_ was achieved using magic-angle spinning (MAS) solid-state NMR spectroscopy.

Solid-state NMR spectroscopy is an atomic-scale technique used to study the dynamics, interactions, and resolve structural complexities and disorders.^39–48^ The focus of this study is to understand PEI impregnation behavior in silica gel, by probing the changes in the different ^1^H, ^13^C environments and the formation and dynamics of the CO_2_ adsorption species at different PEI loadings. Hence, we track changes in the proton and carbon environments of PEI and in the proton environment of H_2_O upon PEI impregnation into silica pores. Furthermore, CO_2_ adsorption was carried out on various PEI/silica samples placed within a solid-state NMR rotor ex-situ. The structure of the resulting CO_2_ adsorption species and the dynamics of these species were investigated using a suite of solid-state NMR experiments and theoretical calculations using Gaussian 6.0.16.^49^

## Materials and Methods

For a systematic study, PEI was used as the amine source, while silica gel was utilized as the silica support. In this study, a range of samples from 20 to 60 weight percent (wt %) of PEI is prepared and systematically analyzed. The successful synthesis of the PEI/silica composite was confirmed using characterization techniques like PXRD, SEM, TGA, FTIR, and nitrogen adsorption isotherm measurements.

### Synthesis of PEI/Silica Composites

#### Materials

Polyethylenimine (branched, molecular weight = 800, obtained from Sigma-Aldrich), Silica (Silica gel, 60 pore size, Sigma-Aldrich), Methanol (Fischer Chemical, 99.9%).

#### Synthesis

The synthesis of PEI/silica was conducted through solvent-assisted impregnation, in accordance with the established procedure.^50^ In a standard synthesis procedure, a precise weight percentage of PEI is carefully measured in a 50 mL beaker. This is followed by thorough mixing with 10 mL of methanol using a magnetic stirrer. Subsequently, an appropriate weight percentage of silica (BET surface area of 487.5 m^2^/g with pore diameter of 6.6 nm, Table S2) is accurately weighed and mixed with the solution. The mixture is allowed to evaporate at room temperature while being continuously stirred. Upon complete evaporation of the solvent, the resultant material is dried in a vacuum oven at 60 °C for 12 h. After the drying process, the material is collected, stored in a glovebox under controlled atmosphere, and prepared for subsequent characterization. The sample code used for different compositions of PEI/silica is explained in Table S1.

### Characterization Techniques

#### Powder X-ray Diffraction (PXRD)

The X-ray diffraction studies were carried out at room temperature using a Malvern Panalytical Empyrean 3 PXRD spectrometer with Cu Kα lamp source for irradiation (λ = 1.5406 Å) in the 2θ range of 10°–60°.

#### Infrared Spectroscopy (ATR-FTIR)

The INVENIO-S Fourier-transform infrared Spectrometer was used in transmittance mode.

#### Nitrogen Adsorption Measurements

Nitrogen adsorption measurements were carried out at liquid nitrogen temperature using nitrogen as a probe molecule with a Micromeritics 3Flex instrument.^51^ A detailed experimental procedure is given in the Supporting Information.

#### Scanning Electron Microscopy (SEM)

The SEM images were obtained by spreading the sample onto carbon tape. Thermo FEI Apreo 2 SEM imaging was performed using Optiplan mode to collect SEM images. EDS spectra (5 kV, 1.6 nA) were collected using Pathfinder software. The high-resolution image is taken with an Acc-voltage of 5 kV and 25 pA.

#### Thermogravimetric Analysis (TGA)

Five to 20 mg of samples were transferred to open alumina crucibles and heated at 10 °C min^–1^ under nitrogen from 30 to 800 °C, using the Netzsch TG 209 F1 Libra instrument. Using the NETZSCH Proteus Thermal Analysis 8.0.2, differential thermogravimetric curves were obtained.

#### Solution-State NMR

^1^H and ^13^C NMR were measured on a 500 MHz Bruker Avance DPX spectrometer using D_2_O as solvent.

#### Solid-State NMR

All solid-state NMR spectra were recorded with a Bruker Avance HD 600 WB NMR spectrometer (ν_0_(^1^H) = 600.5 MHz). The sample was packed into a 3.2 mm rotor, spun at 24 kHz for proton NMR, and for the CO_2_ adsorption experiments, a Kel-F cap (20 kHz) was used. The detailed experimental procedure is given in Section 1 of the Supporting Information.

#### Theoretical Calculations

The solid-state NMR chemical shifts were calculated using Gaussian 16.0.6 software on a high-performance computing (HPC) cluster.^49^ CO_2_ adsorption species were first modeled and optimized via DFT calculations employing the CAM-B3LYP functional and the 6–311++G­(d,p) basis set. The optimized structures were subsequently used for NMR chemical shift predictions using the same basis set. Isotropic chemical shifts obtained from Gaussian calculations were scaled.

## Results and Discussion

### Three Different Cases of PEI Impregnation into Silica Pores

Based on the literature and the various possibilities for the impregnation of PEI at all weight percentage (wt %) loadings in silica, we propose three distinct cases regarding PEI impregnation (Figure 1):^35,38^ (I) Agglomeration: This case posits that PEI agglomerates and does not enter the pores of silica at any concentration. (II) Pore Filling–Agglomeration: In this scenario, PEI enters the pores of silica at lower loadings, and as the concentration increases, PEI impregnates into the silica network. However, at high concentrations, PEI begins to surface agglomerate. (III) Agglomeration–Pore Filling: This case represents the reverse of the second. Here, at lower PEI concentrations, PEI exhibits surface agglomeration, whereas as the concentration increases, PEI impregnates into the silica networks and ultimately enters the pores.

**Figure 1:**
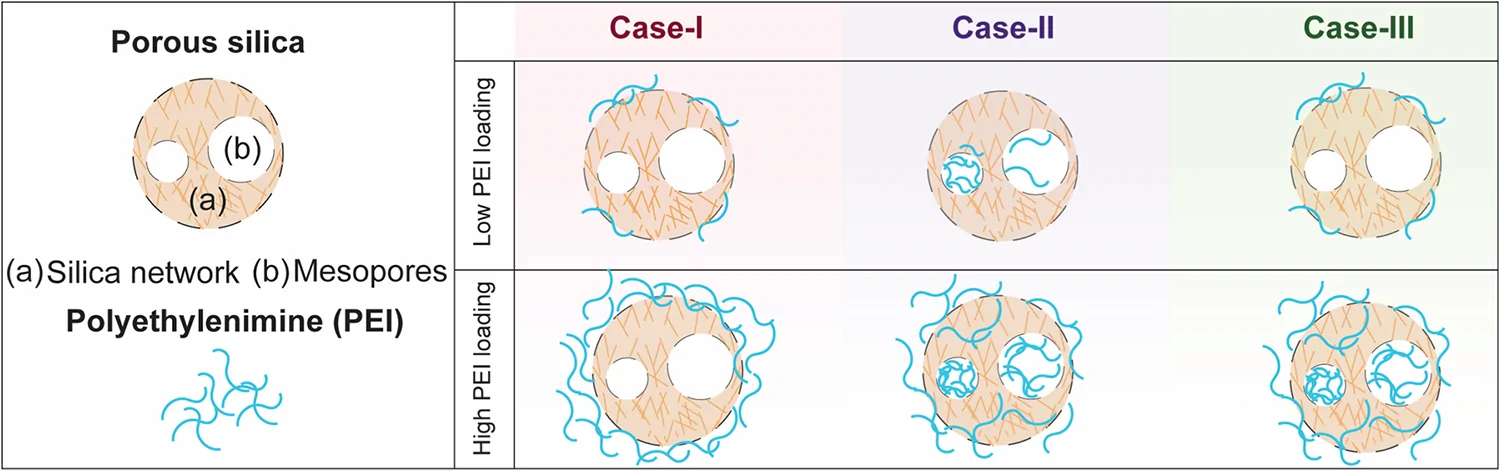
Schematic illustration of PEI impregnation cases in silica composites: Case-I: PEI agglomerates on the surface at all concentrations; Case-II: PEI enters pores at low concentrations but agglomerates at higher ones; Case-III: PEI does not enter pores at low loading but does so at higher loading.

### Insights into Three Different Cases

The PXRD analysis was performed to evaluate the structural stability of silica following the impregnation process (Figure S1). The samples exhibited an amorphous nature, as evident by the PXRD spectra.^52^ A broad peak observed at approximately 22° is characteristic of the silica.^53,54^

The Fourier Transform Infrared Spectroscopy (ATR-FTIR) was utilized to understand the chemical interactions of PEI within the pores of silica. The resulting spectra are presented in Figure S2. For all composites, all the vibrational modes of –NH_2_ (3272, 3343, 1460, and 1566 cm^–1^), Si–OH stretch (958 cm^–1^), and Si–O–Si vibrations (1067 cm^–1^, 797 cm^–1^) are observed in the spectra.^31^ The spectra do not provide conclusive evidence regarding whether the PEI has effectively immobilized within the silica pores following impregnation.

Adsorption isotherm measurements were performed to evaluate the surface area, pore volume, and pore size distribution of various PEI-loaded samples. The adsorption isotherm of precursor silica and all the PEI/silica composites are given in Figure S3. The BJH (Barrett, Joyner, and Halenda) pore size distribution is illustrated in Figure 2a. The surface area, pore volume, and pore size distribution are illustrated in Table S2. The isotherm displays a Brunauer type IV pattern, demonstrating the areas of the monolayer to multilayer adsorption, capillary condensation, and multilayer adsorption.^55^ The presence of a monolayer adsorption region from 0 to ∼0.5 indicates the presence of micropores in silica. This can be advantageous for better transport of CO_2_ adsorption molecules. The average pore diameter of precursor silica was found to be 6.6 nm. The analysis revealed that both the surface area and pore volume of the loaded PEI/silica decreased with an increase in PEI loading, as presented in Table S2. The BET surface area of precursor silica was found to be 487.5 m^2^/g. When impregnated with 20 wt % of PEI, the BET surface area reduced to 274.5 m^2^/g. Furthermore, as the wt % of PEI loading increases, the BET surface area is found to decrease. The same trend is followed in the case of pore volume (Figure 2a and Table S2). As expected, the pore diameter increases with PEI loading, because PEI initially occupies the smaller pores, leaving behind larger pores that are subsequently accessed by N_2_ molecules during BET measurements.^56^ The observations from BET measurements strongly suggest that PEI is effectively impregnating the silica pores rather than merely accumulating on the surface.

**Figure 2:**
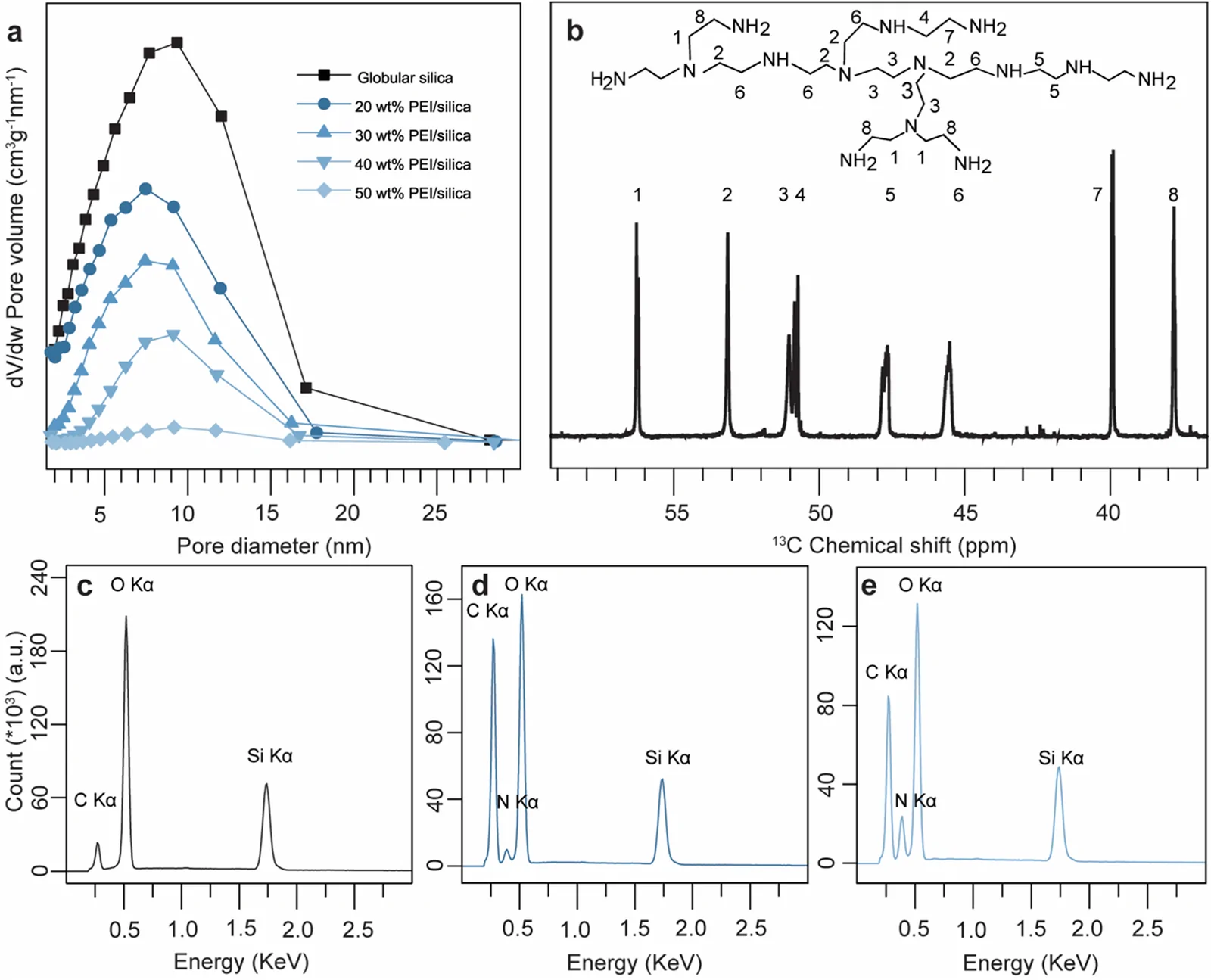
Characterization of PEI-impregnated silica composites. (a) BJH adsorption pore size distribution for all synthesized PEI-impregnated silica composites, derived from nitrogen adsorption isotherm (Faas correction, Harkins and Jura). (b) ^13^C solution state NMR spectra of PEI (in D_2_O solvent) and corresponding carbon peak assignments of PEI. (c–e). Energy dispersive X-ray spectroscopy (EDS) spectra of precursor silica, 20 and 40 wt % PEI silica composites, respectively.

Scanning electron microscopy (SEM) images were acquired to examine the presence of agglomeration of PEI at the silica surface. Figure S4a illustrates the pores of the precursor silica sample, while Figure S4b depicts the 50 wt % PEI/silica sample. The remaining samples are shown in Figure S5. At higher concentrations, PEI forms a viscous layer that leads to agglomeration on the particle surface, as evidenced by Figure S4b. Furthermore, the energy dispersive X-ray spectroscopy (EDS) analysis of the precursor silica, along with the 20 and 40 wt % samples, is presented in Figure 2c–e and the element mapping in Figure S5. It is evident from Figure S5 that as the PEI concentration increases, the silica signal in both the color map and EDS spectra decreases. It should be noted that EDS primarily scans only a few layers at the surface. Similarly, the nitrogen signal in the EDS spectra increases with higher PEI concentrations, becoming significant after 40 wt % PEI loading.

PEI was characterized by solution-state NMR spectroscopy to facilitate the assignment of peaks observed in the corresponding solid-state NMR spectra. The ^1^H and ^13^C spectra, recorded, are illustrated in Figures S6 and 2b, respectively. The ^1^H spectra revealed a series of peaks within the range of 2.25 to 3.25 ppm, attributable to the −CH_2_– backbone protons.^57–59^ The ^13^C spectra were utilized to assign all potential carbon environments present in the PEI structure, as depicted in Figure 2b.^57–59^ This characterization will serve as a basis for the peak assignments in the subsequent analysis of the ^13^C solid-state NMR spectra.

Based on observations derived from PXRD, FT-IR spectroscopy, and nitrogen adsorption isotherm measurements, the agglomeration case (case I in Figure 1) is less likely. The observed reduction in surface area with increasing PEI loading indicates that PEI is not agglomerating on the silica surface. If agglomeration were occurring at all concentrations of PEI loading, the BET isotherms would appear similar; however, this is not the observed case. The available spectroscopic evidence is currently insufficient to confirm the possibility of cases II and III. To elucidate this, it is essential to examine the molecular-level interactions and noncovalent interactions within the composites. Alterations in the proton water environments in silica upon the incorporation of PEI can be studied utilizing solid-state NMR spectroscopy. Furthermore, CO_2_ adsorption occurs at the solid–gas interface, which necessitates the use of solid-state NMR for the thorough examination of this material.

### Insights from Solid-State NMR Experiments

The ^1^H MAS NMR spectra of precursor silica were collected at 24 kHz of magic angle spinning speed (Figure S7). Three sets of peaks were observed in the ^1^H spectra at 1.06, 3.56, and 5.01 ppm. These peaks are arising from isolated surface hydroxyl groups on silica at 1.06 ppm, isolated water molecules that do not interact with the surface at 3.56 ppm, and water molecules that are hydrogen-bonded to −OH groups at 5.01 ppm, respectively.^60,61^ The ^1^H MAS NMR spectra of the PEI/silica composites at different loadings are collected and illustrated in Figure 3 (the spectra are detailed in Figure S8). The chemical shift assignments of relevant peaks are given in Table 1. In pure silica, the adsorbed H_2_O exhibits a resonance at 5.01 ppm, consistent with values reported in the literature.^60,61^ Upon PEI impregnation, the peak at 5.01 ppm shifts to 5.95 ppm in the 20 wt % PEI-loaded sample (Figure 3 and Table 1). And this shift can be because of the interaction between the water molecules and the impregnated PEI. With an increase in the concentration of PEI loading, the peak shifted to 5.51 ppm for 40 wt % loading and 4.80 ppm for 60 wt % loading (Figure 3). This phenomenon indicates that the H_2_O environment within silica is altered by PEI impregnation (Figure 3), suggesting that case II (Figure 1) is possible. In addition to that, as the PEI concentration increases, the ^1^H peaks are getting narrower. Buntkowsky and co-workers have systematically examined the behavior of water environments in mesoporous silica materials, concluding that there exists a direct correlation between the dynamics of water protons and the line shape and chemical shift changes of ^1^H solid-state NMR spectra.^62^ A similar trend is observed in the ^1^H MAS NMR spectra of the PEI/silica composites used in this study. Although these results indicate that the ^1^H environments are affected by PEI impregnation, further experiments and analysis are required to elucidate changes in the mobility of H_2_O, chemical exchange of water protons, and hydrogen-bonding interactions upon PEI incorporation.

**Figure 3:**
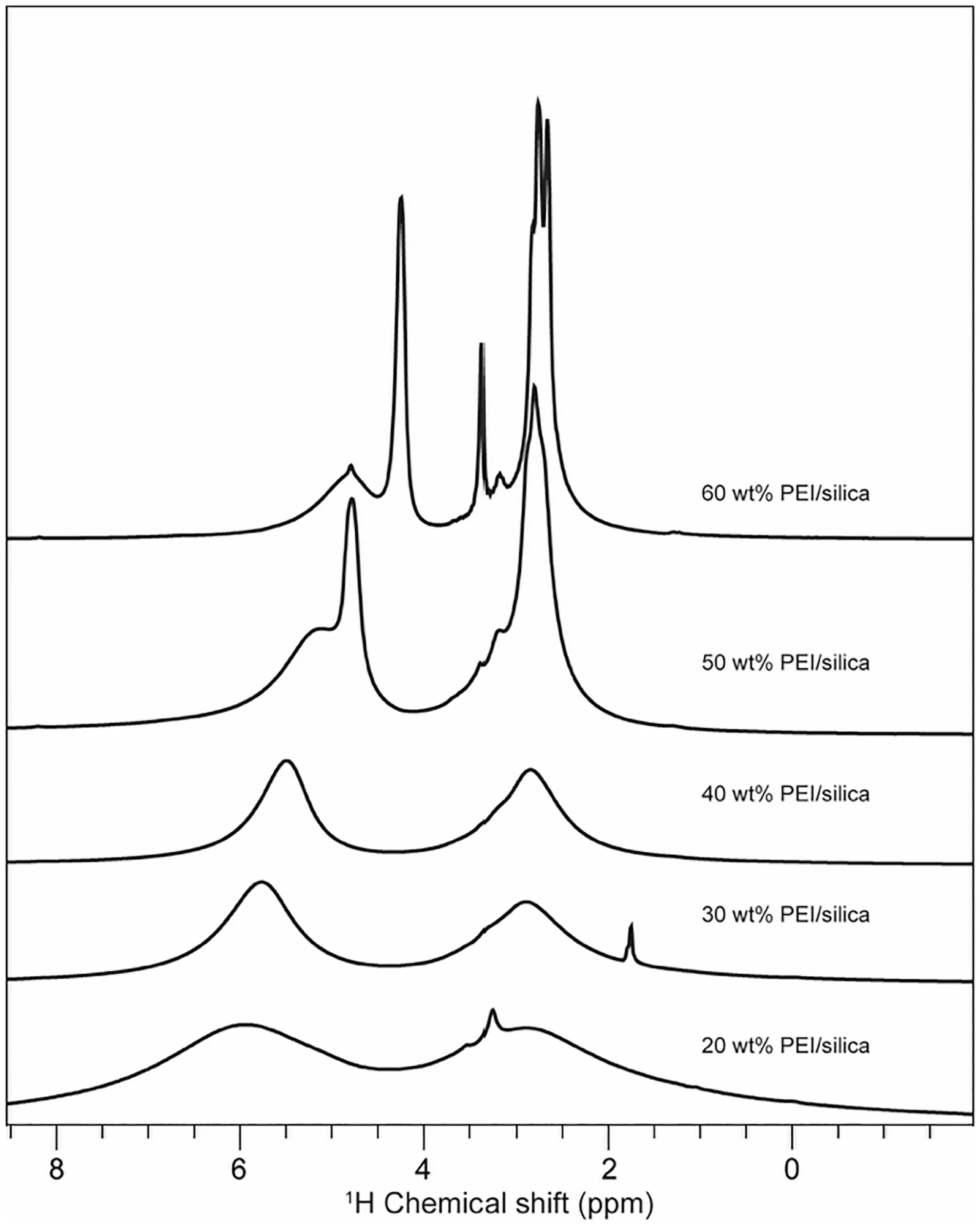
Solid-state ^1^H NMR spectra of 20, 30, 40, 50, and 60 wt % PEI silica composites recorded at 24 kHz spinning speed.

**Table 1:** Solid-State ^1^H NMR Peaks of PEI/Silica Samples with Different PEI Loading are Compared with the Literature Value

literature value^60,61^	pure silica	20 wt % PEI/silica	30 wt % PEI/silica	40 wt % PEI/silica	50 wt % PEI/silica	60 wt % PEI/silica
1.7	1.06	^a^	1.77	^a^	^a^	^a^
3.8	3.56	2.88	2.92	2.87	2.81^b^	2.82, 2.75, 2.66
5.4	5.01	5.95	5.78	5.51	5.18^c^, 4.79	4.8^c^, 4.23

a Difficult to resolve the peaks.

b With shoulder peaks.

c Broad peak | the peaks overlap at higher concentrations.

The peaks appearing in the 1.0 to 3.6 ppm originate from the PEI backbone and the free water within the pores. At higher PEI loadings (60 wt %), multiple peaks become resolved, and the spectra closely resemble the solution state NMR spectra of PEI. These additional resonances arise from the agglomeration of PEI on the silica surface at higher concentrations. This trend is consistent with case II. The ^1^H–^13^C cross-polarization magic angle spinning (CPMAS) NMR spectra for the PEI/silica composites at a spinning speed of 24 kHz are illustrated in Figure S9. At lower concentrations, the peaks are broad; however, at significantly higher concentrations, the peaks become narrower and more distinct. This behavior indicates a change in the dynamics of the polymer as the loading of PEI varies. At lower loading, the mobility of PEI is restricted due to the strong interaction with the silica pore walls and silica network, resulting in broader peaks. In contrast, at very high concentrations, dynamics leads to a narrowing of the spectra. Furthermore, at higher PEI concentrations, all carbon environments are distinctly observable, similar to those depicted in solution NMR in Figure 2b. This observation further substantiates the validity of case II. A two-dimensional ^1^H–^13^C heteronuclear correlation (HETCOR) spectrum was acquired for 40 wt % PEI/silica at a spinning speed of 24 kHz prior to CO_2_ adsorption (Figure S10). As anticipated, the backbone carbons of PEI are clearly resolved. The broadening of ^1^H–^13^C CP spectra and ^1^H MAS NMR at 20, 30, and 40 wt % of PEI/silica composites suggests significant restrictions in the dynamics of PEI. This phenomenon can be attributed to the strong interactions between PEI and the silica pore walls as well as the silica networks. At lower concentrations, the polymer is capable of forming a conformal layer that exhibits robust interactions with the silica surfaces.^35,38^ Conversely, at elevated concentrations, the surface agglomeration of PEI on the silica surfaces induces dynamic behavior, resulting in peak narrowing. It can be concluded that the pores and networks of silica become saturated with PEI following a loading of 40 wt %. This is evident from the drastic decrease in BET surface area and pore volume after 40 wt % PEI loading (Table S2), which is further corroborated by the accompanying changes in the solid-state NMR line shapes.

### Ex-Situ CO_2_ Adsorption Studies

The thermogravimetric analysis (TGA) of the PEI/silica samples before and after CO_2_ adsorption was carried out (Figure S11). Two major mass losses, at approximately 120 and 370 °C, were observed. The mass loss occurring near 120 °C is attributed to the desorption of physisorbed water.^63^ The mass loss observed near 370 °C corresponds to the thermal decomposition of PEI. For both mass losses, a clear trend is observed: the magnitude of the mass loss increases with increasing PEI content.

CO_2_ adsorption experiments of 20, 40, and 60 wt % samples were conducted using a 3.2 mm solid-state NMR rotor. The detailed experimental procedure is provided in Section 2 of the Supporting Information. Prior to CO_2_ adsorption, a vacuum (0.1 mbar) was applied to remove any loosely bound H_2_O molecules from the rotor with the PEI/silica composite and the adsorption setup.

The ^1^H MAS NMR spectra of 40 and 60 wt % PEI/silica samples before and after CO_2_ adsorption are shown in Figure S12a,b, respectively. For the 40 wt % composite, the spectrum broadened upon CO_2_ adsorption. This enhanced broadness is attributed to increased water cluster formation, facilitated by multiple hydrogen bonding interactions with CO_2_ adsorption species.^64^ In contrast, the ^1^H NMR spectra of 60 wt % PEI/silica composite show no remarkable change after the CO_2_ adsorption. Figure 4a represents the ^1^H–^13^C CP spectra of 40 wt % PEI/silica before and after CO_2_ exposure (additional spectra are provided in Figure S13). The ^13^C resonance near 164 ppm is assigned to the formation of carbamate species.^65,66^ However, given the presence of moisture in the sample, the possibility of carbonate/bicarbonate formation cannot be ruled out. Nevertheless, the higher amine concentration, together with supporting literature evidence, suggests that carbamate species are more likely to form.^28,65,67^ The peak at 164 ppm is prominent for 20 and 40 wt % PEI loading. However, in the 60 wt % composite, the peak intensity is significantly reduced (Figure 4b). This can be due to extensive pore blockage (case II—Figure 1) at higher PEI loading, which blocks CO_2_ diffusion into the silica pores and, consequently, prevents interaction with the active sites. Similar pore blockage at elevated PEI loadings in mesoporous materials has been reported previously.^68–70^

**Figure 4:**
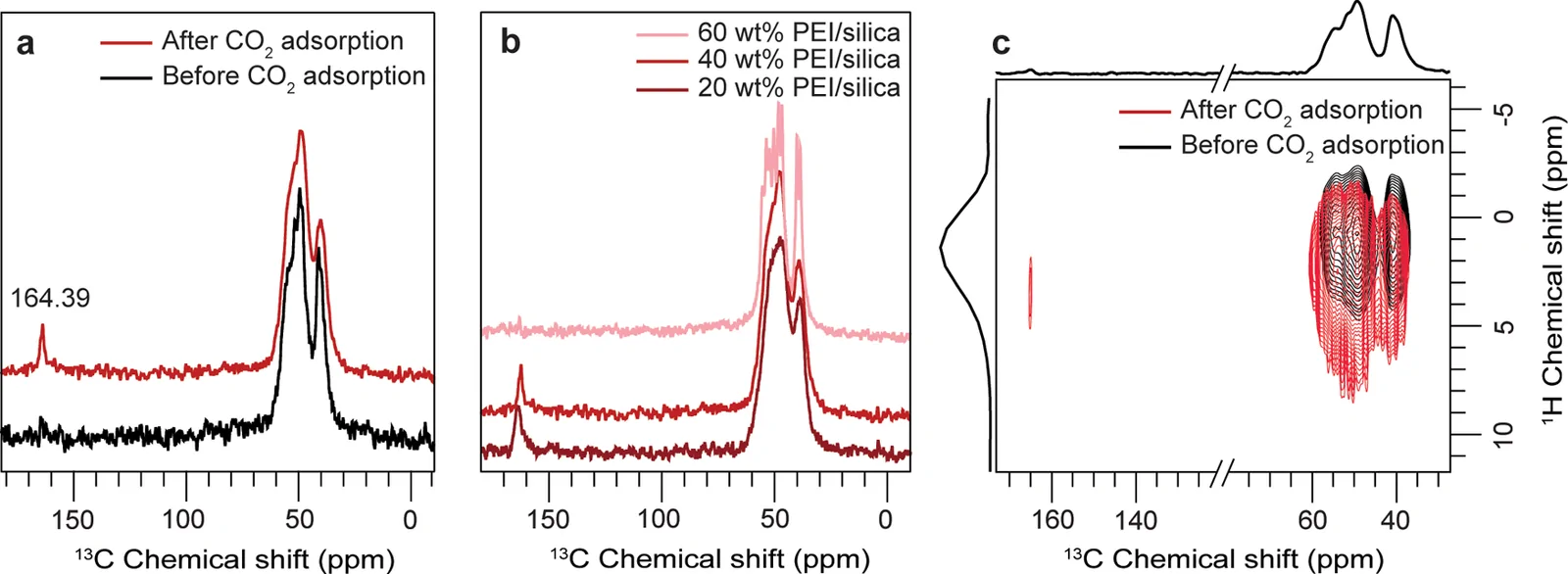
Solid-state NMR spectra recorded at a 20 kHz spinning speed after a CO_2_ adsorption of 1 h. (a) ^1^H–^13^C CP solid state NMR spectra of 40 wt % PEI/silica composites before and after CO_2_ adsorption. (b) A comparison of ^1^H–^13^C CP spectra of 20, 40, and 60 wt % PEI/silica composites after CO_2_ adsorption. (c) The ^1^H–^13^C HETCOR spectra of a 40 wt % PEI/silica composite after CO_2_ adsorption.

The ^1^H–^13^C HETCOR MAS NMR spectra recorded at a 20 kHz spinning speed, both before and after CO_2_ adsorption, are presented in Figure 4c. After CO_2_ uptake, a new correlation peak appears at 164 ppm, consistent with the formation of carbamate species. In addition, changes observed in the ^13^C peaks upon CO_2_ adsorption. A similar observation could be seen in the ^1^H–^13^C two-dimensional HETCOR correlation spectra. These observations provide direct spectroscopical evidence for heterogeneous chemical environments around the amine group following the CO_2_ capture.

The ^1^H–^13^C CP experiments were recorded at varying contact times, and the one with the shortest contact time gave the maximum signal intensity (Figure S14). With longer contact, spin diffusion might be prominent, resulting in a reduction in signal intensity.

To gain theoretical insight into the experimental findings, two CO_2_ adsorption products, carbamate species, were modeled, and chemical shift predictions were performed.^60,66^ The geometry optimization resulted in a carbamic acid species (Figure S15 and Table S3). The carbonyl carbon of the carbamic acid appears approximately at 164 ppm. The predicted chemical shift using Gaussian software is also in the same line. However, in this theoretical modeling, only the carbamate species was modeled and did not explicitly account for solvent effects, confinement within the pores, or additional stabilization arising from, for example, surrounding water molecules. This level of modeling was sufficient to support the experimental chemical shift. Nevertheless, a more accurate description would require including all the above-mentioned effects, which we propose as a direction for future work.

## Conclusions

In this study, we studied molecular-level noncovalent interactions during the impregnation of PEI into silica pores. We synthesized PEI/silica composites with 20, 30, 40, 50, and 60 wt % PEI for systematic analysis. The composites were characterized by PXRD, nitrogen adsorption/desorption isotherms, TGA, SEM-EDS, IR, and solution-state NMR.

Solid-state NMR experiments constitute a unique technique for probing noncovalent interactions in PEI/silica composite. In this study, we examined the molecular-level interactions between silica and PEI in the presence of H_2_O and CO_2_. The CO_2_ adsorption studies were performed across several PEI loadings in a systematic way, and the primary adsorption product was identified. Theoretical modeling and chemical shift prediction also support this observation. Based on the literature and the complementary characterization techniques such as PXRD, FTIR spectroscopy, and nitrogen adsorption isotherm, three possible scenarios describing the interaction between silica and PEI are discussed. The case I was ruled out based on the observations from nitrogen adsorption measurements, SEM-EDS, etc. A detailed comparison of the ^1^H and ^13^C NMR spectra confirmed that the Case II accurately represents the system. The ^1^H–^13^C CP NMR spectra show broad carbon resonance for the aliphatic carbons at lower PEI loadings. The broad peaks in CP are due to strong interactions between PEI and silica pores at low concentrations. Once the PEI loading exceeds 40 wt %, the pores become saturated, and PEI begins to agglomerate on the external surface. However, the CO_2_ adsorption process was conducted ex situ, and structural rearrangement or redistribution of adsorbed species may have occurred during transfer from the adsorption setup to the NMR probe. These effects were not examined here but could be explored in future work. Overall, the combined solid-state NMR, computational, and complementary characterization results provide a molecular-level picture of PEI/silica interactions and CO_2_ chemisorption. These insights can guide the rational design of improved amine-functionalized adsorbents for CO_2_ capture applications.

Obtaining an atomistic picture of small-molecule interactions (e.g., H_2_O, CO_2_) within porous materials such as MOFs and zeolites remains challenging.^71,72^ Solid-state NMR spectroscopy is sensitive to changes in the chemical environment of probe nuclei. By employing various solid-state NMR experiments and systematically introducing small molecules into porous materials, it is possible to investigate covalent and noncovalent interactions within the material.

## Supplementary Material



## Data Availability

The data supporting the findings of this study are available within the manuscript and its Supporting Information.

## Notes

Additional information on characterization techniques, custom-built gas adsorption setup for solid-state NMR rotor, nitrogen adsorption isotherm, theoretical modeling of CO2 adsorption species, PXRD, FT-IR, SEM_EDS, TGA, solution-state NMR, and detailed solid-state NMR results (PDF)
