# Supplementary material for: Insights into CO2 Adsorption Motifs in Functionalized Mesoporous Silica Using Solid-State NMR
Source: ACS Omega. 2026 Aug 12;11(33):49872–80. doi: 10.1021/acsomega.6c04679 (PMC13520008; doi:10.1021/acsomega.6c04679)
Supplement: Supplementary file 1 [file ao6c04679_si_001.pdf]

Insights into CO_2 Adsorption Motifs in Functionalized Mesoporous Silica Using Solid-State NMR

Mohammed Jasil[ab], Athulya Nadol[b], Hyeseung Jeong[b], Brijith Thomas[ab*]

[a]Department of Chemistry, New York University, New York, New York 10003, United States

[b]Science Division, New York University Abu Dhabi (NYUAD), Abu Dhabi 129188, United Arab Emirates.

Table of contents

1	Instrumental and methodological information on characterization techniques<br>(a) Powder X-ray Diffraction (PXRD)<br>(b) Infrared Spectroscopy (FT-IR)<br>(c) Adsorption Isotherm measurements<br>(d) Solution-state NMR<br>(e) Solid-state NMR<br>(f) TGA	S2
2	Experimental set up and methodology for CO_2 adsorption studies by solid-state NMR	S3
3	Table S1: The description of the sample code used	S4
4	Table S2: The nitrogen adsorption isotherm results detailing the surface area, pore volume, and pore diameter of the precursor globular silica and all PEI/silica compositions	S4
5	Table S3: Theoretically predicted NMR chemical shift for CO_2 adsorption species	S4
6	Figure S1: PXRD	S5
6	Figure S2: FT-IR	S6
7	Figure S3: The nitrogen adsorption isotherm	S7
8	Figure S4: SEM	S7
9	Figure S5: SEM-EDS	S8
10	Figure S6: The ^1H solution-state NMR spectra of precursor PEI	S8
11	Figure S7: Solid-state ^1H NMR spectra of precursor silica	S9
12	Figure S8: Solid-state ^1H NMR spectra of all PEI/silica compositions at 24 kHz spinning speed	S10
13	Figure S9: Solid-state ^{13}C NMR spectra of all PEI/silica compositions at 24 kHz spinning speed	S11
14	Figure S10: Solid-state NMR ^1H-^{13}C correlation HETCOR spectra	S12
15	Figure S11: Thermogravimetric analysis (TGA) before and after CO_2 adsorption	S12
16	Figure S12. Solid-state ^1H NMR spectra recorded before and after CO_2 adsorption on 40 and 60 weight percentage PEI/silica composites	S13

17	Figure S13: Solid-state ^1H - ^{13}C CP NMR spectra recorded after CO_2 adsorption on 20 and 60 weight percentage PEI/silica composites	S13
18	Figure S14. Solid-state ^1H - ^{13}C CP NMR spectra recorded after CO_2 adsorption at different contact times	S14
19	Figure S15: Gaussian optimized structures of bicarbonate and carbamate species	S14
20	References	S15

1. Instrumental and methodological information on characterization techniques

Powder X-ray Diffraction (PXRD):

The X-ray diffraction studies were carried out using a Malvern Panalytical Empyrean 3 PXRD spectrometer with Cu Kα lamp source for irradiation (λ = 1.5406 Å).

Fourier Transform Infrared Spectrometer (ATR-FTIR):

The INVENIO-S Fourier Transform Infrared Spectrometer (IR) was utilized.

Adsorption Isotherm measurements:

The adsorption isotherms were studied using nitrogen as a probe molecule at liquid nitrogen temperature with a Micromeritics 3Flex instrument. The specific surface area measurements were performed using the Brunauer-Emmett-Teller (BET) calculation.[1] The material was degassed at 150 °C for 6 hours to remove adsorbed gas molecules and moisture. The classical BJH (Barrett, Joyner, and Halenda) method determined the pore size distribution (Harkins and Jura thickness equation and BJH analyses with Halsey-Faas correction to derive the pore size information).[2]

Thermogravimetric analysis (TGA): 5 to 20 mg of samples were transferred to open alumina crucibles and heated at 10 °C min-1 under nitrogen from 30 to 800 °C, using the Netzsch TG 209 F1 Libra instrument. Using the NETZSCH Proteus Thermal Analysis 8.0.2, differential thermogravimetric curves were obtained.

Scanning electron microscopy (SEM):

The scanning electron microscopy (SEM) images were obtained by spreading the sample onto carbon tape. Thermo FEI Apreo 2 SEM imaging was performed using Optiplan mode to collect SEM images. EDS spectra (5 kV, 1.6 nA) were collected using Pathfinder software. The high-resolution image is taken with an Acc—voltage of 5kV and 25 pA.

Solution-state NMR:

^1H and ^{13}C NMR in solution were measured on a 500 MHz Bruker Avance DPX spectrometer using D_2O as solvent.

Solid-state NMR:

All solid-state NMR spectra were measured with a Bruker Avance HD 600 WB NMR spectrometer ($\nu_0(^1H)$ = 600.5 MHz). The experimental procedure reported in our lab is followed.[3,4]

Sample spinning at 24 kHz (3.2 mm rotor):

The sample was loaded into a 3.2 mm ZrO_2 rotor (spinning at 24 kHz for proton NMR), and for the CO_2 adsorption experiments, a Kel-F cap (spinning at 20 kHz) was used. For all 1D and 2D experiments, 24 kHz was used as the spinning speed. The ^1H-^{13}C ramp-cross polarization (rampCP)[5] experiments were performed with a ^{13}C nutation frequency of 60 kHz, and the ^1H nutation frequency optimized at the +1 Hartman-Hahn condition[6] ~84 kHz. The repetition delay was set to 2 s. $^1H\{^{13}C\}$ CP-HETCOR experiments were performed with a contact time of 2500 μs and 500 μs. DUMBO[7] homonuclear decoupling schemes were employed during the t1 evolution at ~100 kHz. The ^1H and ^{13}C scaling factor for DUMBO HETCOR was corrected using adamantine as the standard. The ^1H spectra were normalized with respect to the peak around 5 ppm. We checked the reproducibility of the NMR data by synthesizing the material under identical conditions. The ^1H and ^{13}C chemical shifts were reproduced with uncertainties of ±0.3 and ±0.7 ppm, respectively. The trends in peak widths and changes in chemical shifts as a function of PEI loading were also reproducible.

The longitudinal relaxation delay of samples was assessed utilizing the saturation recovery pulse sequence method through the T_1/T_2 Relaxation program by Bruker. This method involved fitting the data to an exponential equation to obtain accurate results:

$I = I_0 + Pe^{((-\tau/T_1))}$

I is the intensity of the resonance at the inversion delay time τ and I_0 at the equilibrium state, and P is a constant.

2. Experimental setup and methodology for CO_2 adsorption studies by solid-state NMR

The CO_2 adsorption experiment was conducted using an apparatus similar to that depicted below. A three-necked, flat-bottomed, round-bottom flask (RB flask) capable of withstanding up to 2 bar gas pressure was custom-designed. One minor neck was connected to a vacuum gauge, while another was linked to a Schlenk line. The Schlenk line's gas channel was attached to a CO_2 cylinder, and its vacuum channel was connected to a high-vacuum pump via a digital vacuum meter and cold trap. Prior to the experiment, the entire system underwent repeated evacuation cycles. Additionally, the Dewar flask of the cold trap was filled with liquid nitrogen.

A 3.2 mm solid-state NMR rotor was packed with the sample inside a glove box, as the synthesized samples for CO_2 adsorption studies were stored under inert conditions. The uncapped rotor was carefully placed on the solid support within the RB flask. The system was sealed and subjected to further cycles of evacuation. Subsequently, CO_2 gas was introduced via the Schlenk line and maintained at 1 bar for 1 hour, facilitating diffusion of CO_2 into both the sample in the NMR rotor.

Upon completion, the rotor was removed, immediately sealed with a Kel-F cap, and transferred directly into the NMR probe for measurement at a spinning rate of 20 kHz.

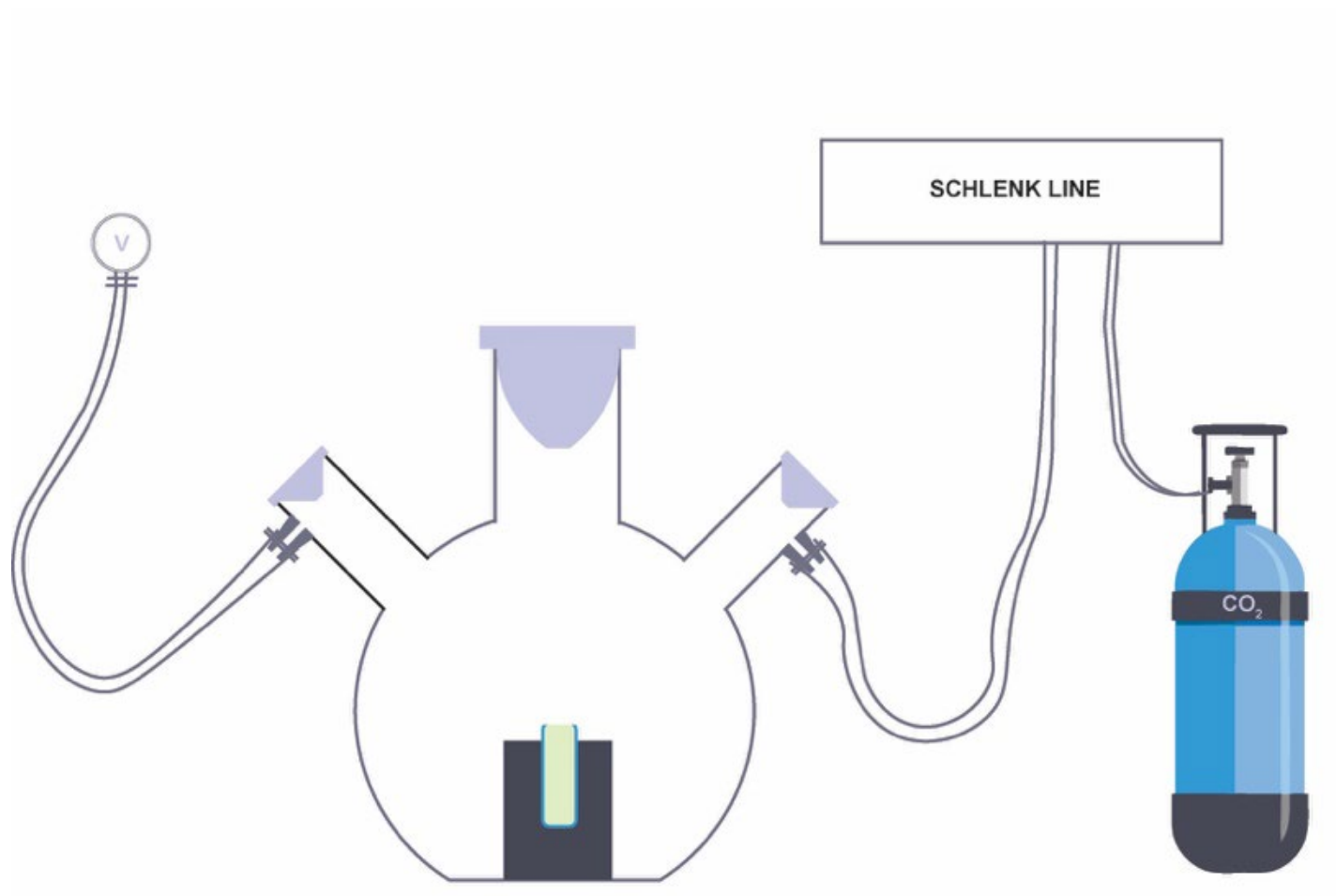

Table S1: The description of the sample code used.

Concentration of PEI used (weight %)	Concentration of globular silica used (weight %)	Sample code for composite
20	80	20 wt% PEI/silica
30	70	30 wt% PEI/silica
40	60	40 wt% PEI/silica
50	50	50 wt% PEI/silica
60	40	60 wt% PEI/silica

Table S2: The nitrogen adsorption isotherm results detailing the surface area, pore volume, and pore diameter of the precursor globular silica and all PEI/silica compositions are summarized below.

Sample	Surface area (m^2/g)	Pore volume (cm^3/g)	Pore diameter (nm)
Precursor silica	487.5	0.81	6.62
20 wt% PEI/silica	274.53	0.48	6.94
30 wt% PEI/silica	148.65	0.30	8.17
40 wt% PEI/silica	62.16	0.15	9.41
50 wt% PEI/silica	9.49	0.02	9.23
60 wt% PEI/silica	0	0	-

Table S3: Theoretically predicted NMR chemical shift for the CO_2 adsorption product obtained using Gaussian 6.0.16.[8] Isotropic chemical shift obtained from Gaussian calculations were scaled.

CO_2 adsorption species	^{13}C predicted chemical shift
Carbamic acid 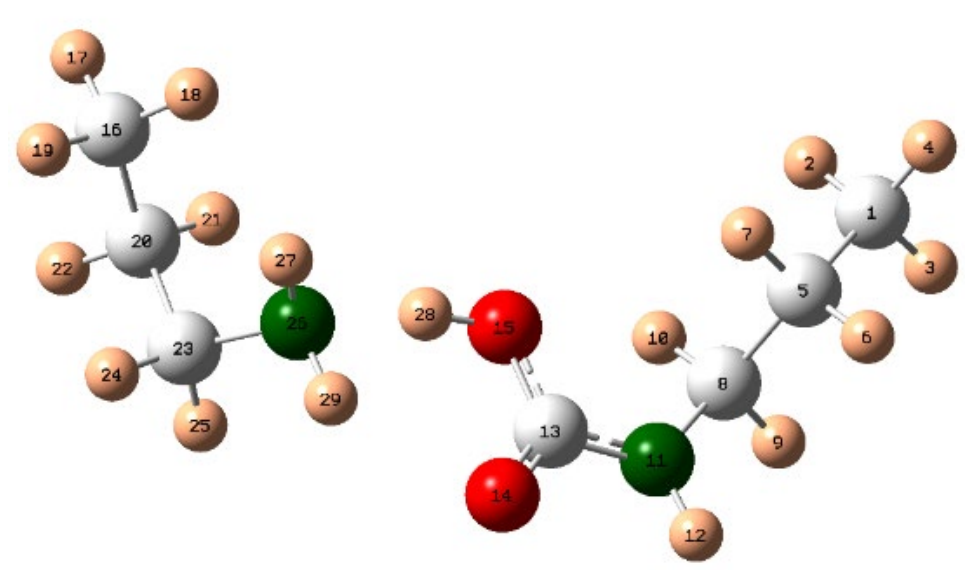	167 ppm

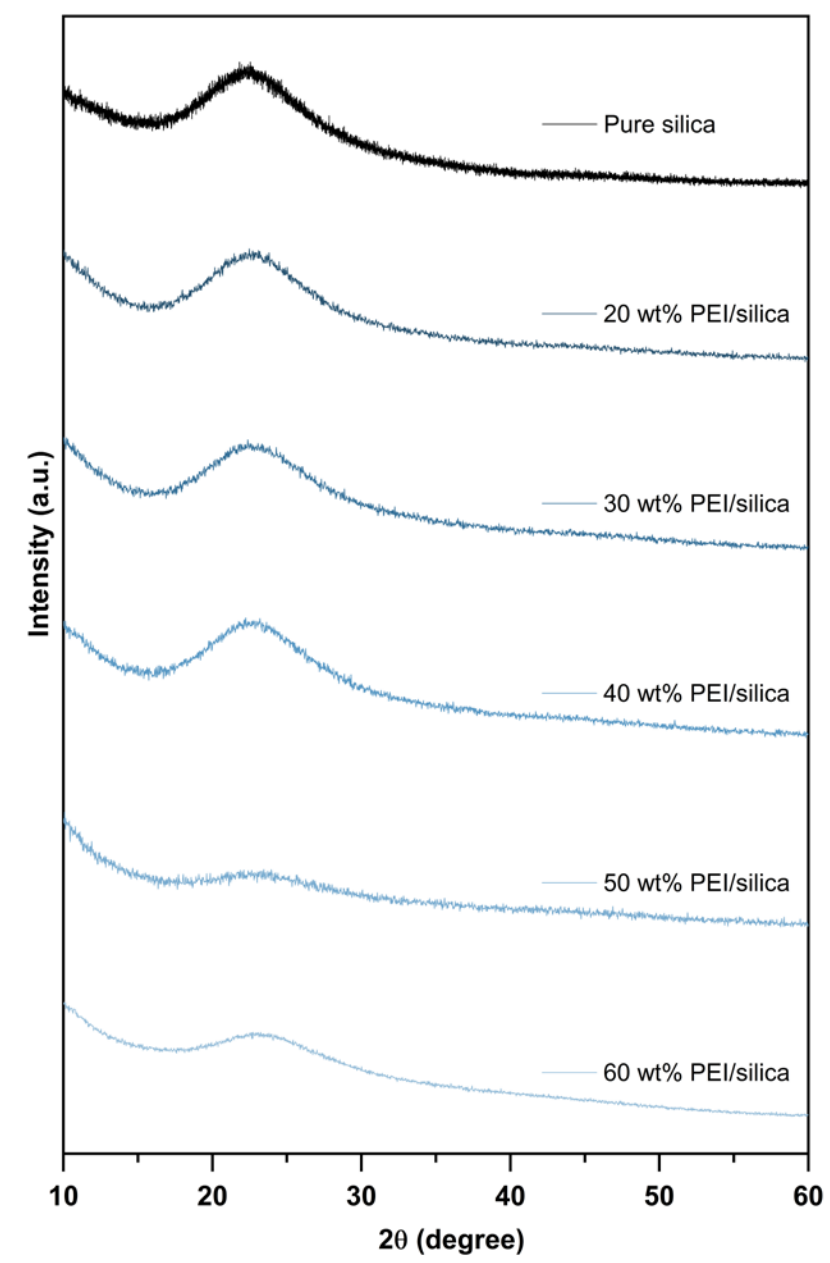

Figure S1. Powder X-ray diffraction patterns of precursor globular silica and composites with 10 to 60 weight percentage PEI.

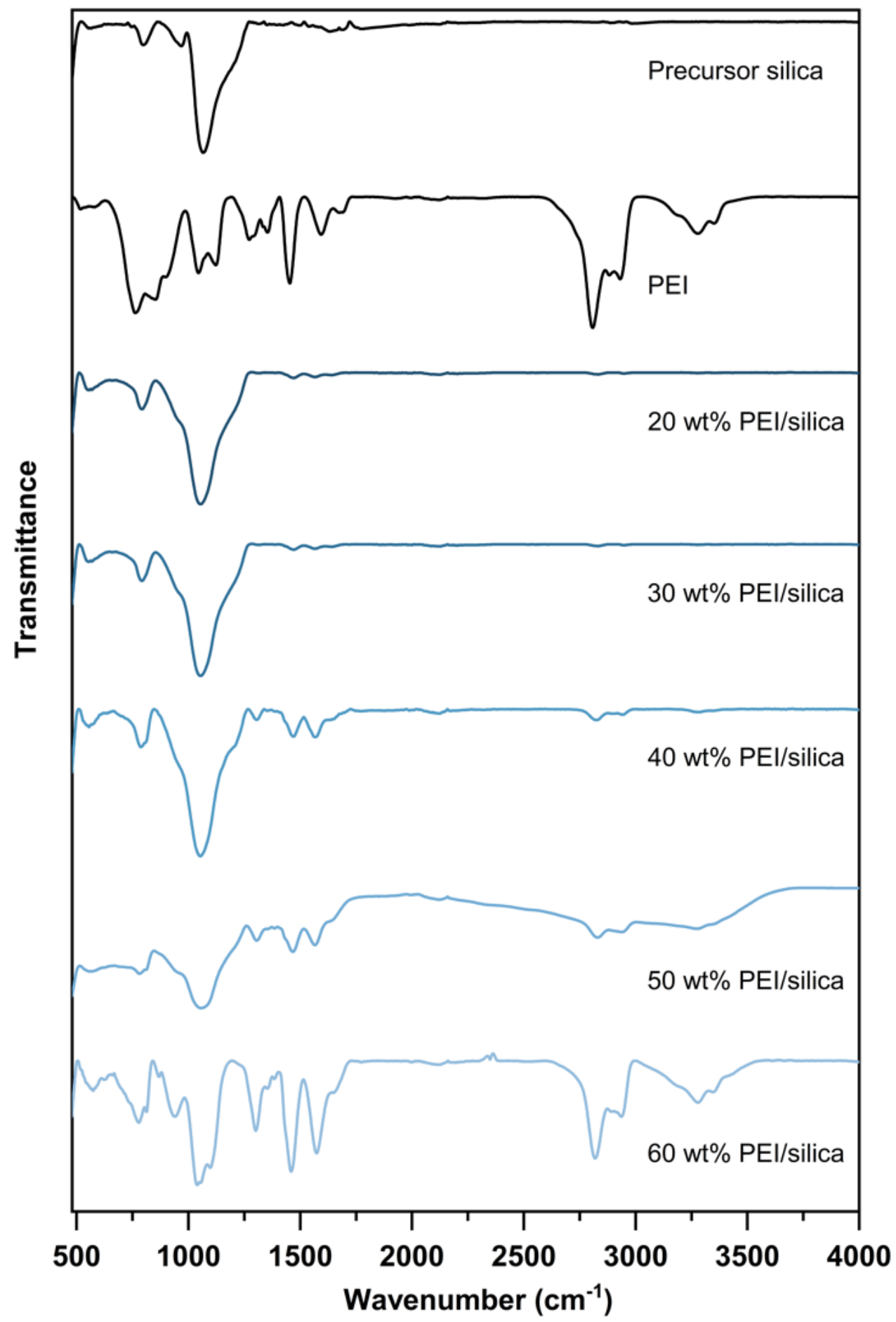

Figure S2. The infrared (FT-IR) spectrum of globular silica and PEI-impregnated silica composites, ranging from 10 wt% to 60 wt% PEI with a molecular weight of 800.

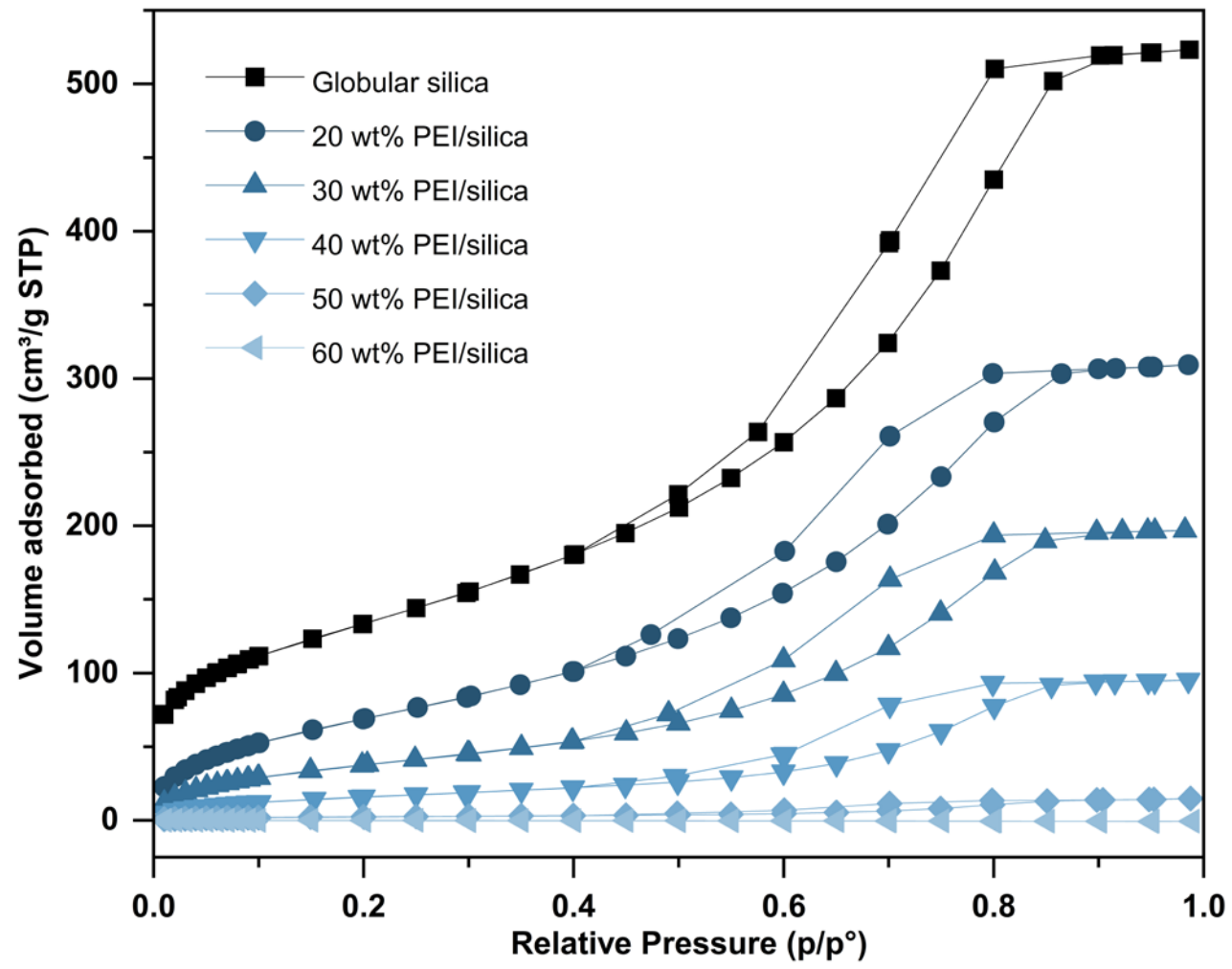

Figure S3. The nitrogen adsorption isotherm of synthesized PEI-impregnated silica samples (volume of N_2 adsorbed at liquid nitrogen temperature against relative pressure, at liquid nitrogen temperature, and N_2 molecule as the probe molecule).

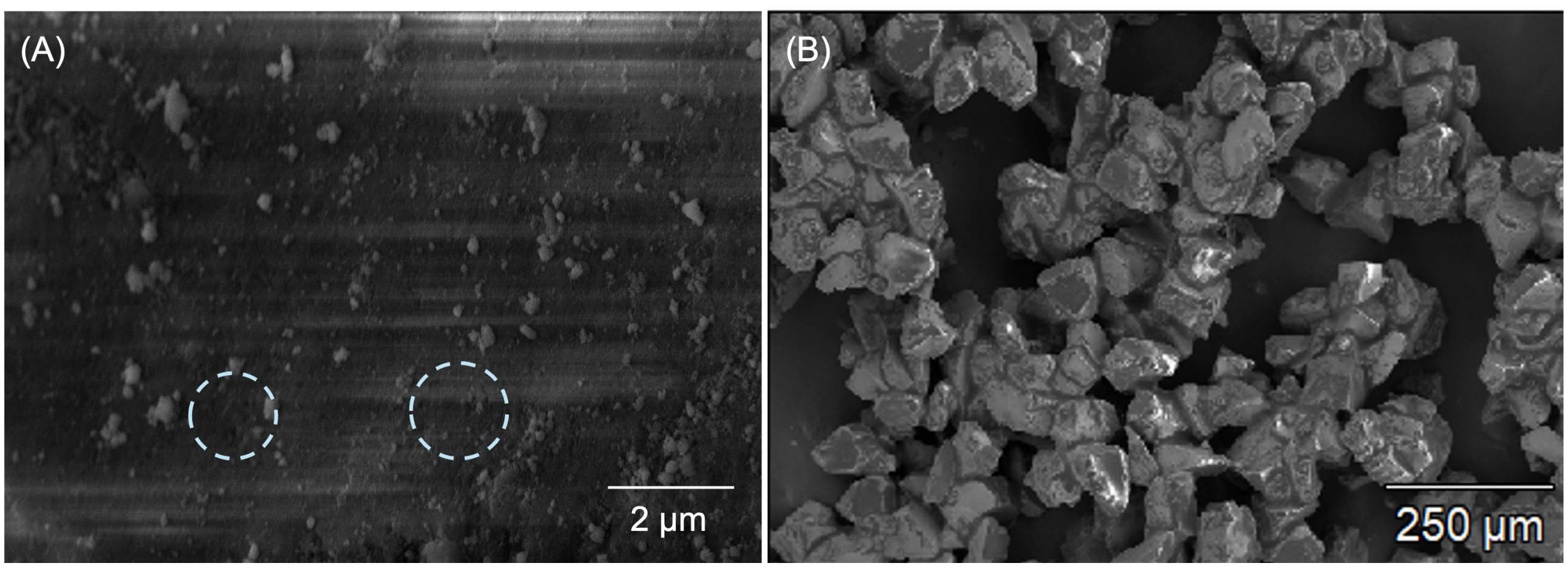

Figure S4. (a) SEM image of precursor silica surface with the pores at the surface (some of the pores are circled in the figure). (b) SEM image of 50 wt% PEI silica sample.

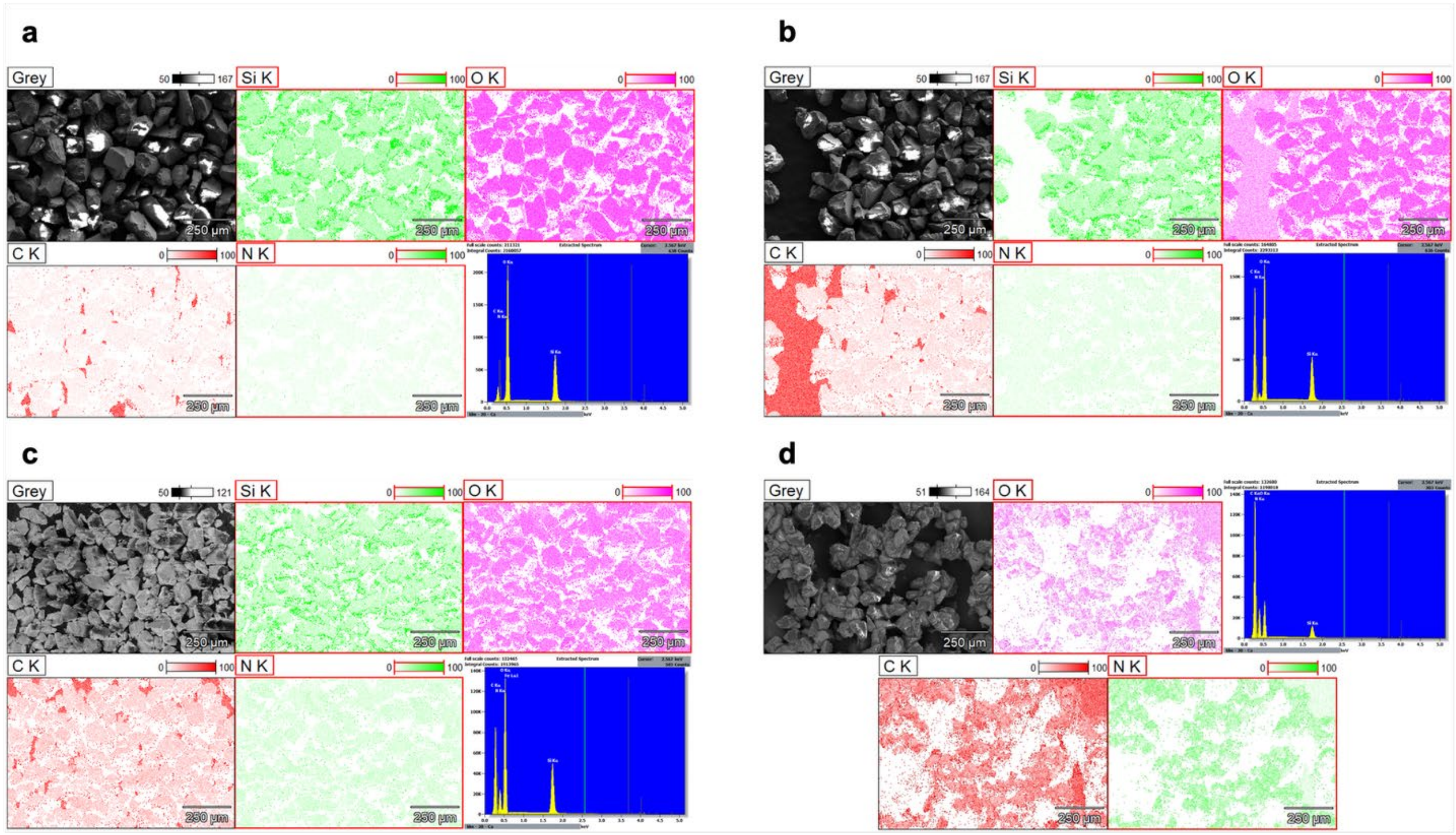

Figure S5. SEM-EDS colour map images of (a) precursor silica, (b) 20 wt% PEI/silica, (c) 40 wt% PEI/silica, and (d) 50 wt% PEI/silica

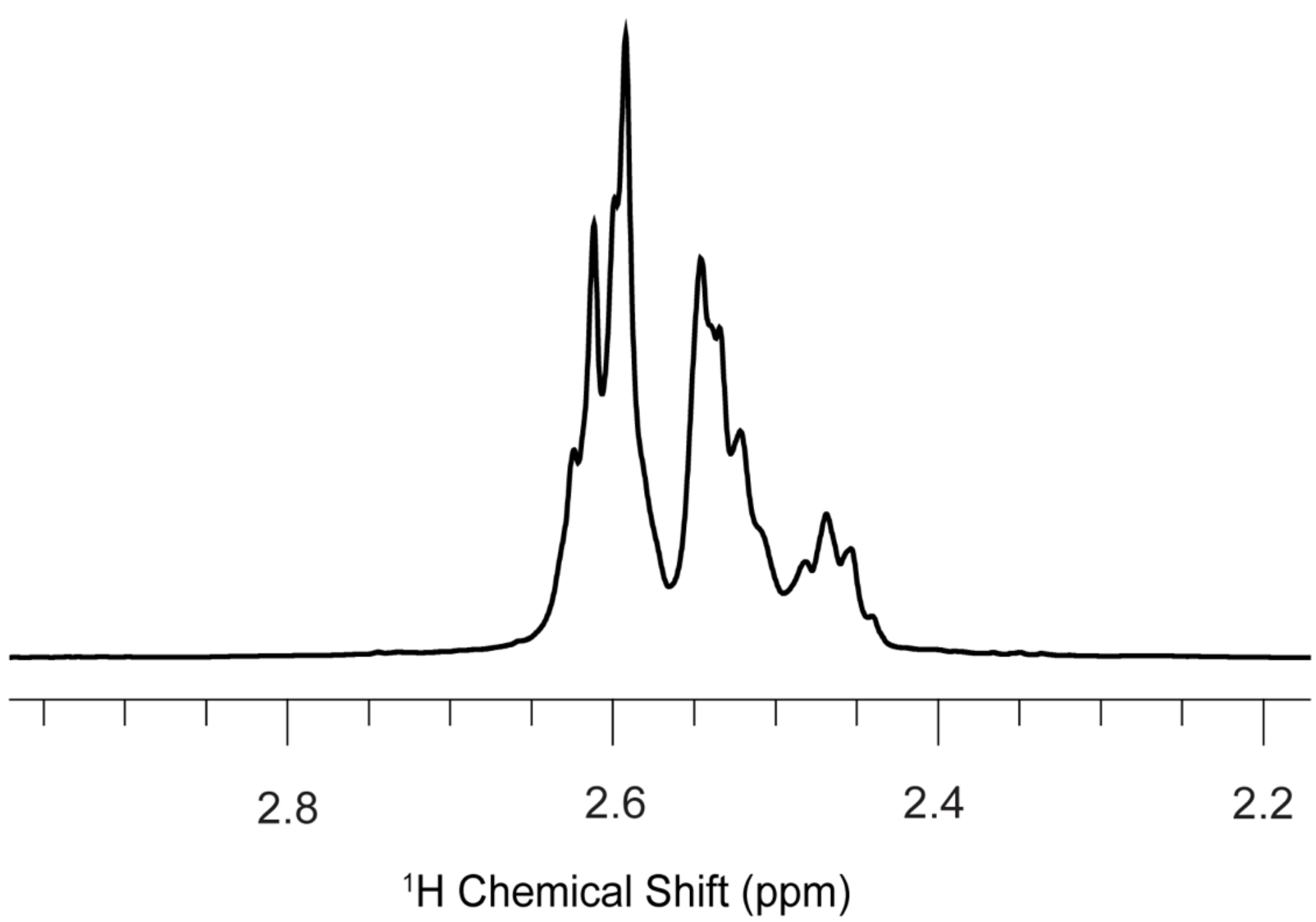

Figure S6. The ^{1}H solution-state NMR spectra of precursor PEI.

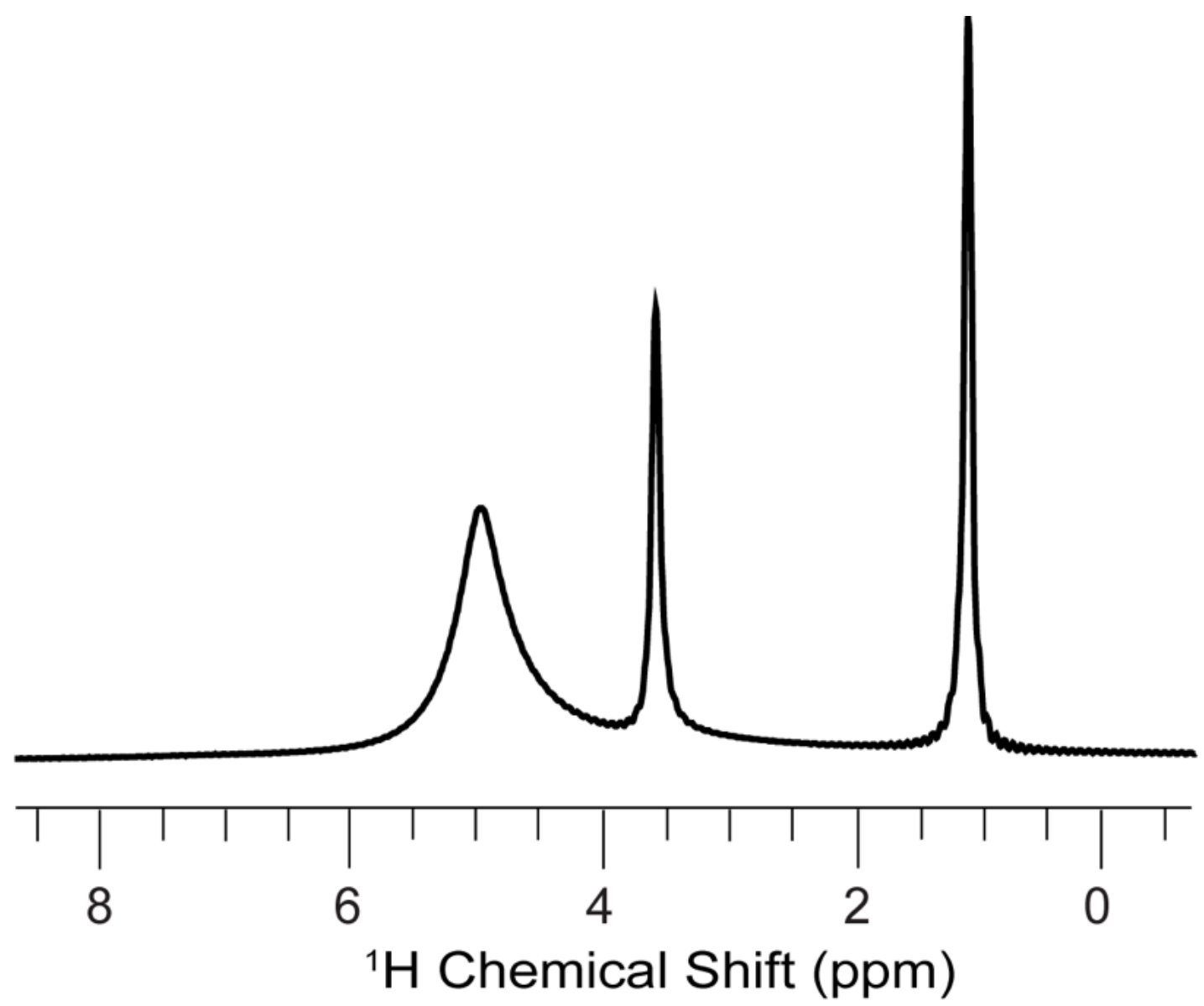

Figure S7. Solid-state ^{1}H NMR spectra of precursor globular silica at 24 kHz spinning speed.

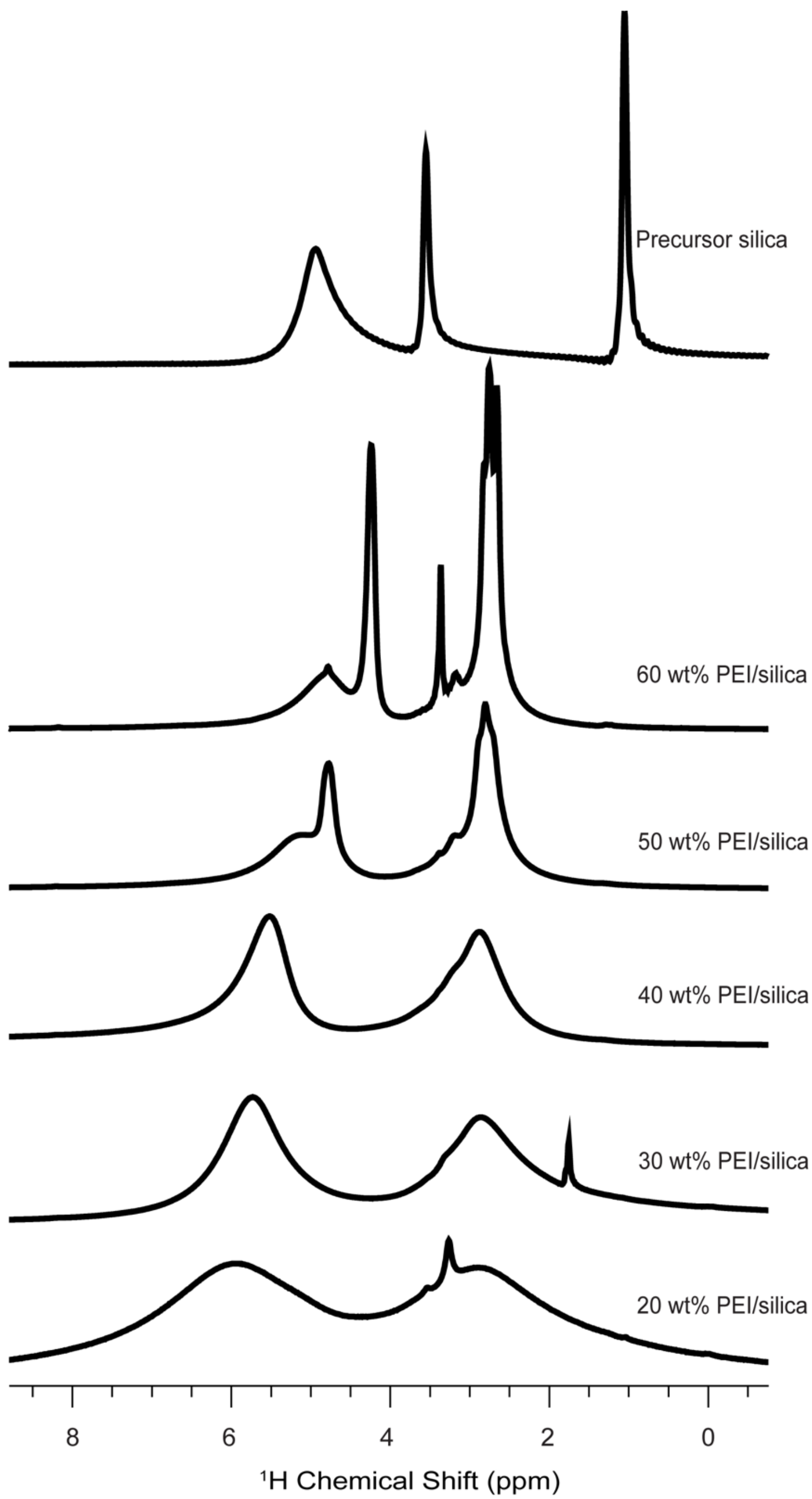

Figure S8. Solid-state ¹H NMR spectra of all PEI/silica compositions at 24 kHz spinning speed.

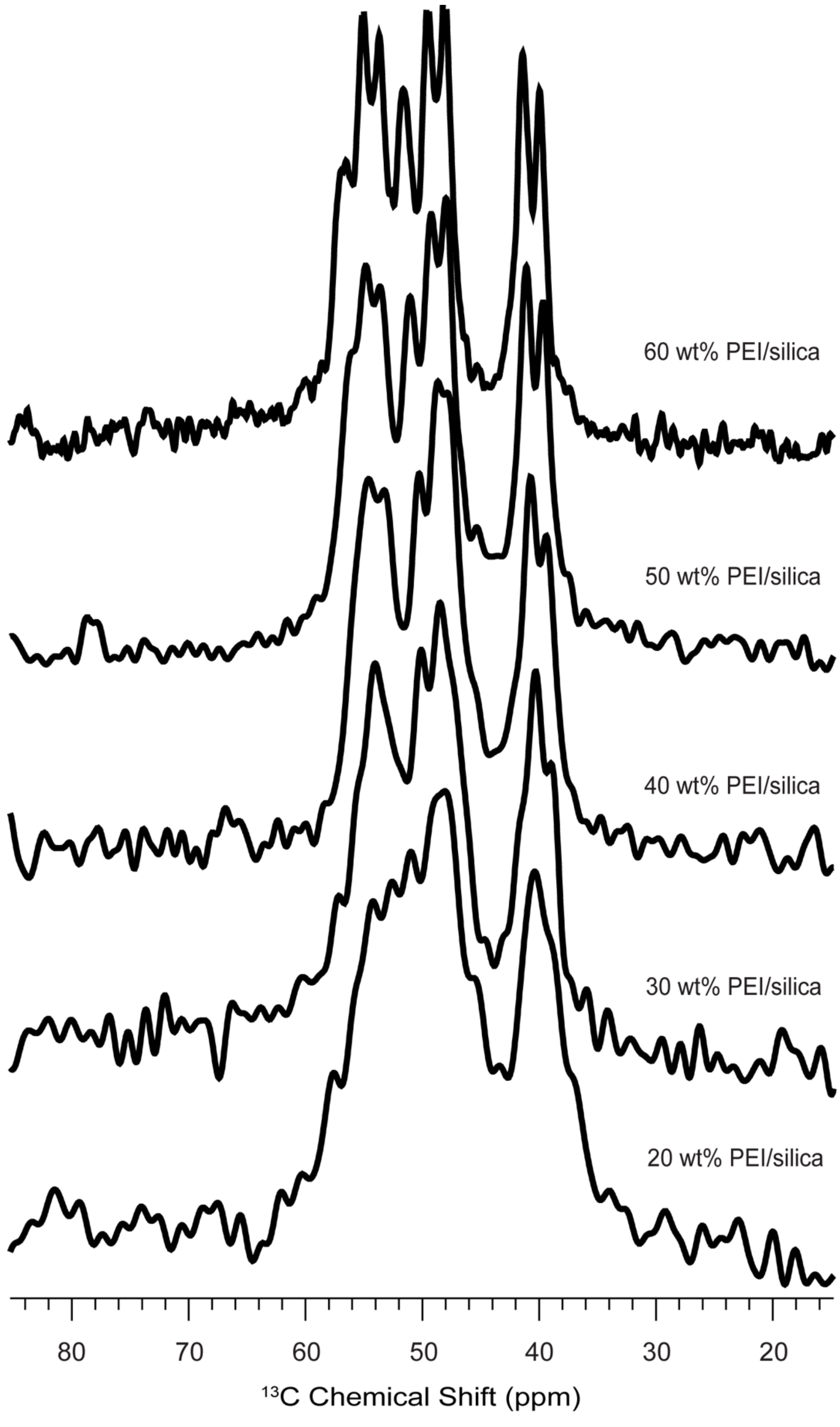

Figure S9. Solid-state ^{13}C NMR spectra of all PEI/silica compositions at 24 kHz spinning speed.

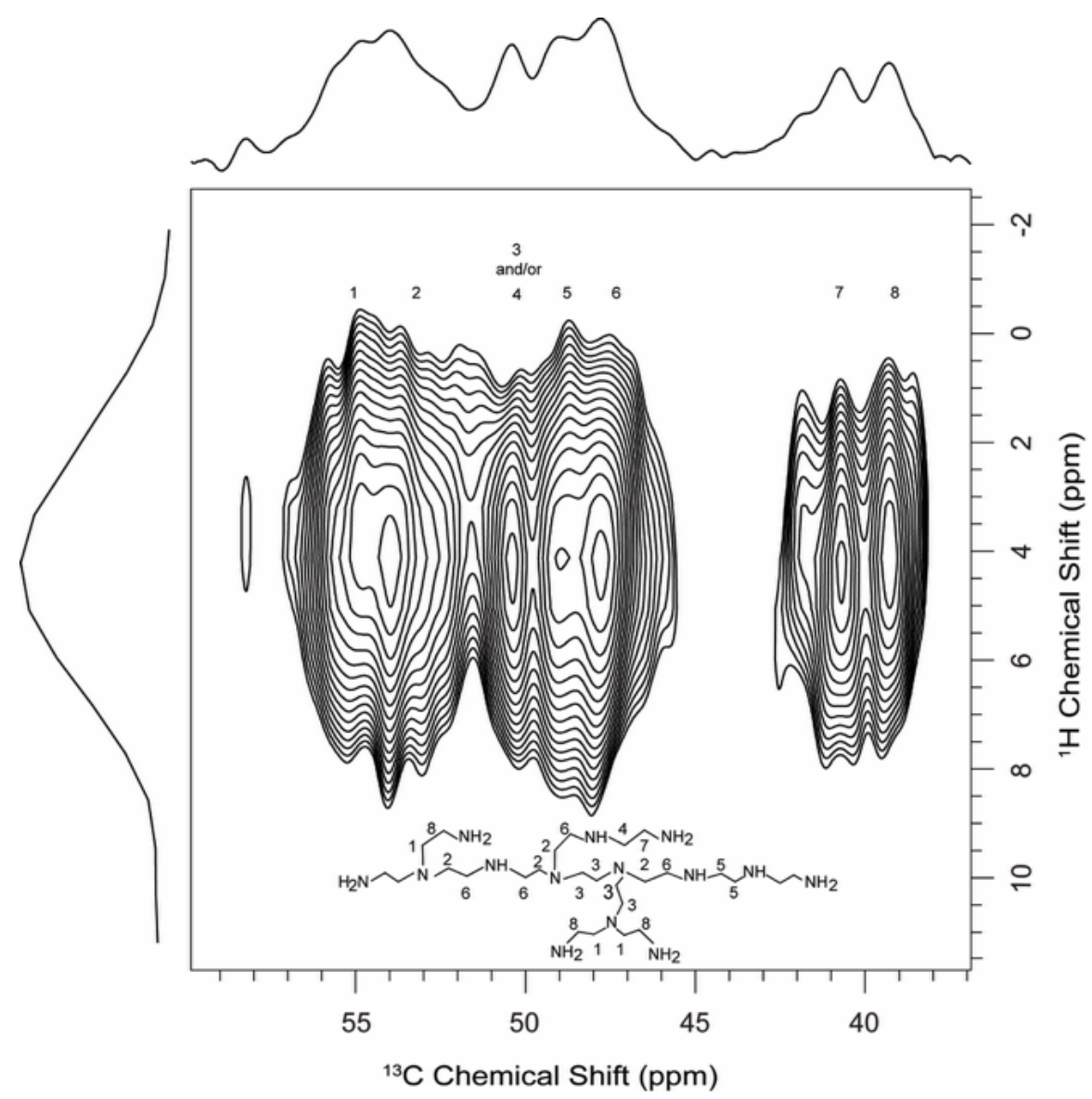

Figure S10. ^1H–^{13}C correlation HETCOR spectra (at 24kHz spinning) of 40 wt% PEI impregnated globular silica showing the correlations between carbon and protons of polymer backbone at a short contact time of 500 μs.

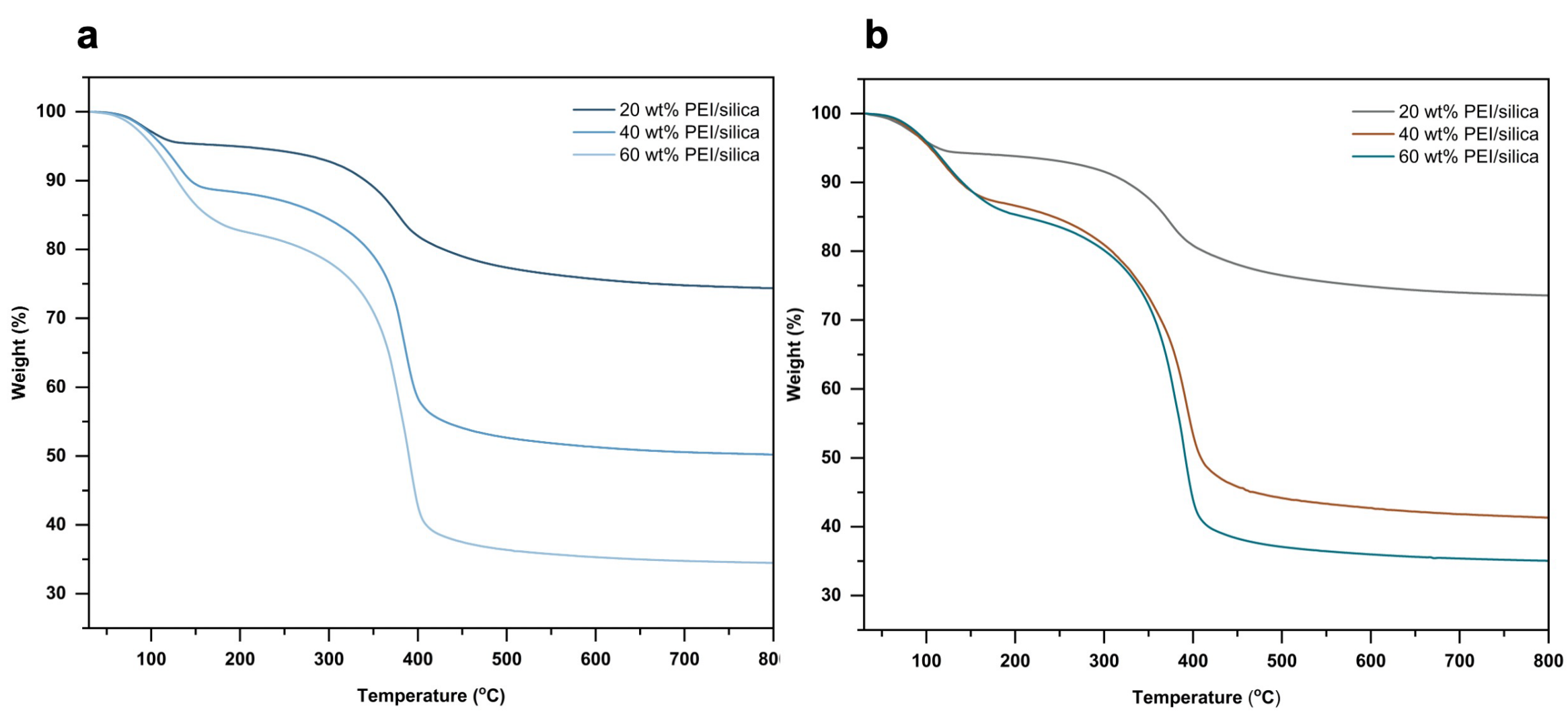

Figure S11. Thermogravimetric curves (TGA) were measured for the 20, 40, and 60 weight percentages of PEI/silica. (a) before CO_2 exposure between 30 °C and 800 °C and (b) after CO_2 exposure for 60 minutes between 30 °C and 800 °C. Two distinct mass losses are observed around 120 °C and 270 °C.

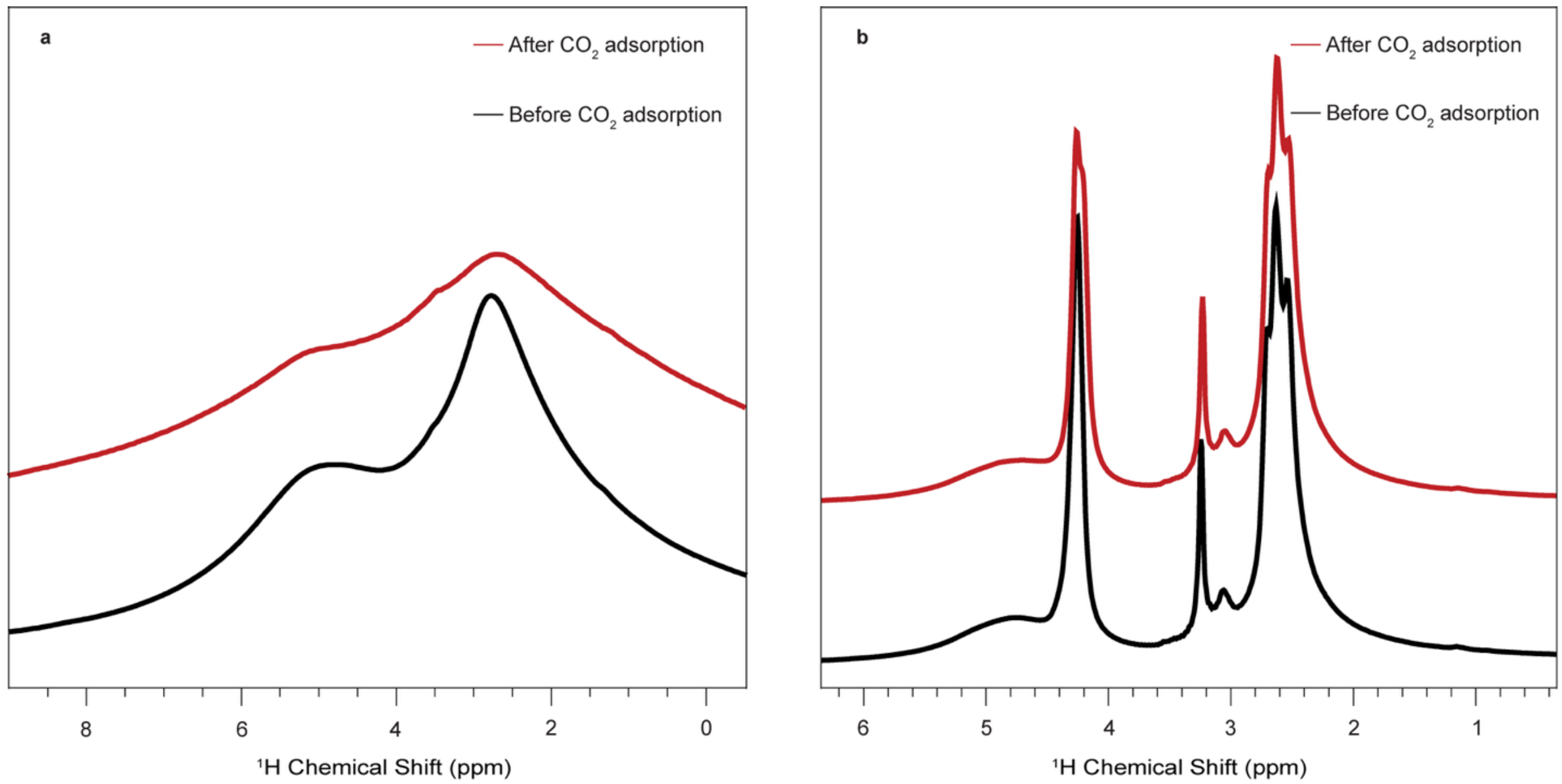

Figure S12. Solid-state NMR spectra recorded at a 20 kHz spinning speed before and after a CO_2 adsorption of 1 hour. (a) Solid-state ^{1}H NMR spectra of 40 weight percentage PEI/silica composites before and after CO_2 adsorption. (b) Solid-state ^{1}H NMR spectra of 60 weight percentage PEI/silica composites before and after CO_2 adsorption.

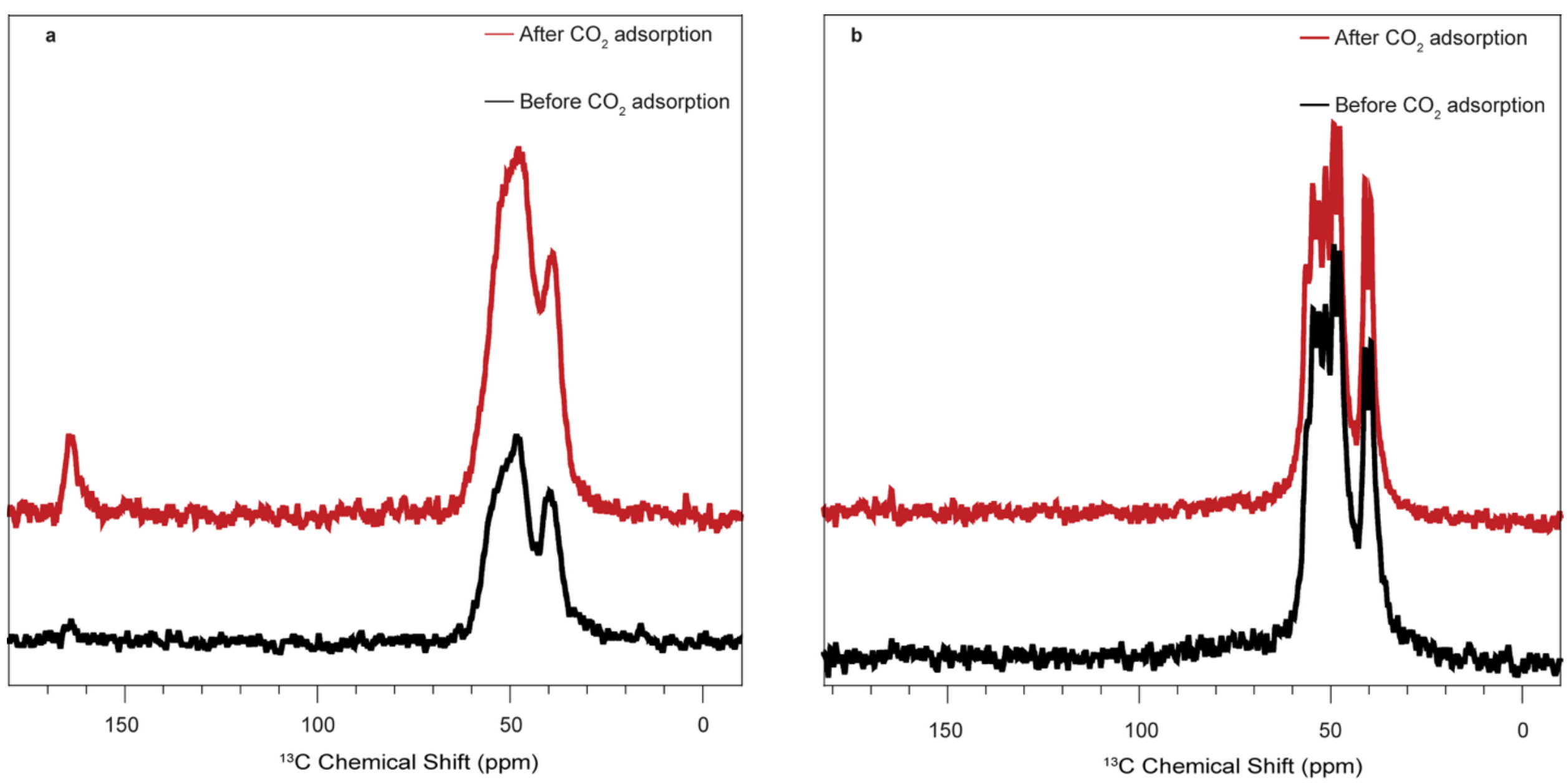

Figure S13. Solid-state NMR spectra recorded at a 20 kHz spinning speed after a CO_2 adsorption of 1 hour. (a) Solid-state ^{1}H - ^{13}C CP NMR spectra of 20 weight percentage PEI/silica composites before and after CO_2 adsorption. (b) Solid-state ^{1}H - ^{13}C CP NMR spectra of 60 weight percentage PEI/silica composites before and after CO_2 adsorption.

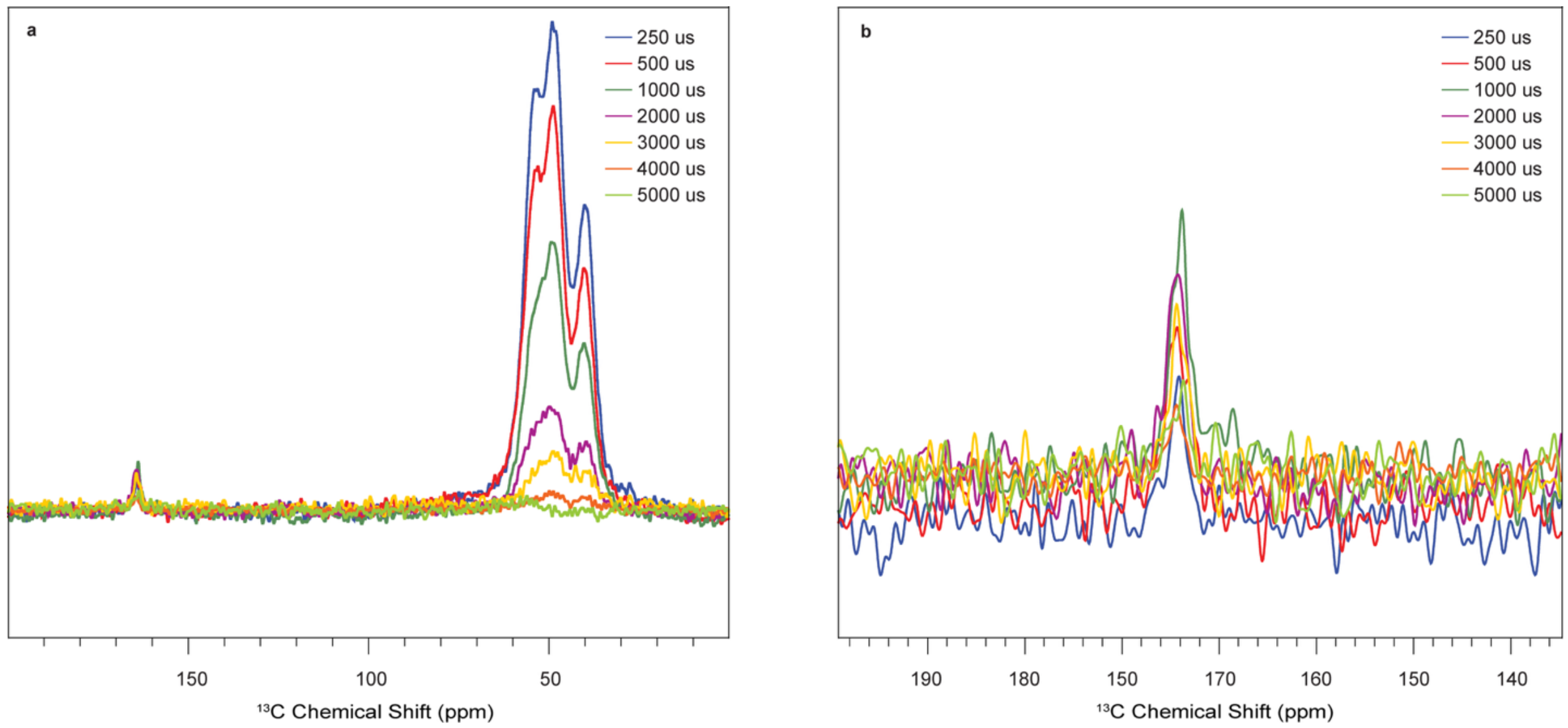

Figure S14. Solid-state ^{1}H - ^{13}C CP NMR spectra of 40 wt% PEI/silica recorded at a 20 kHz spinning speed after CO_2 adsorption at different contact times (the entire spectrum is shown in (a), and the carbamate region is shown in (b)).

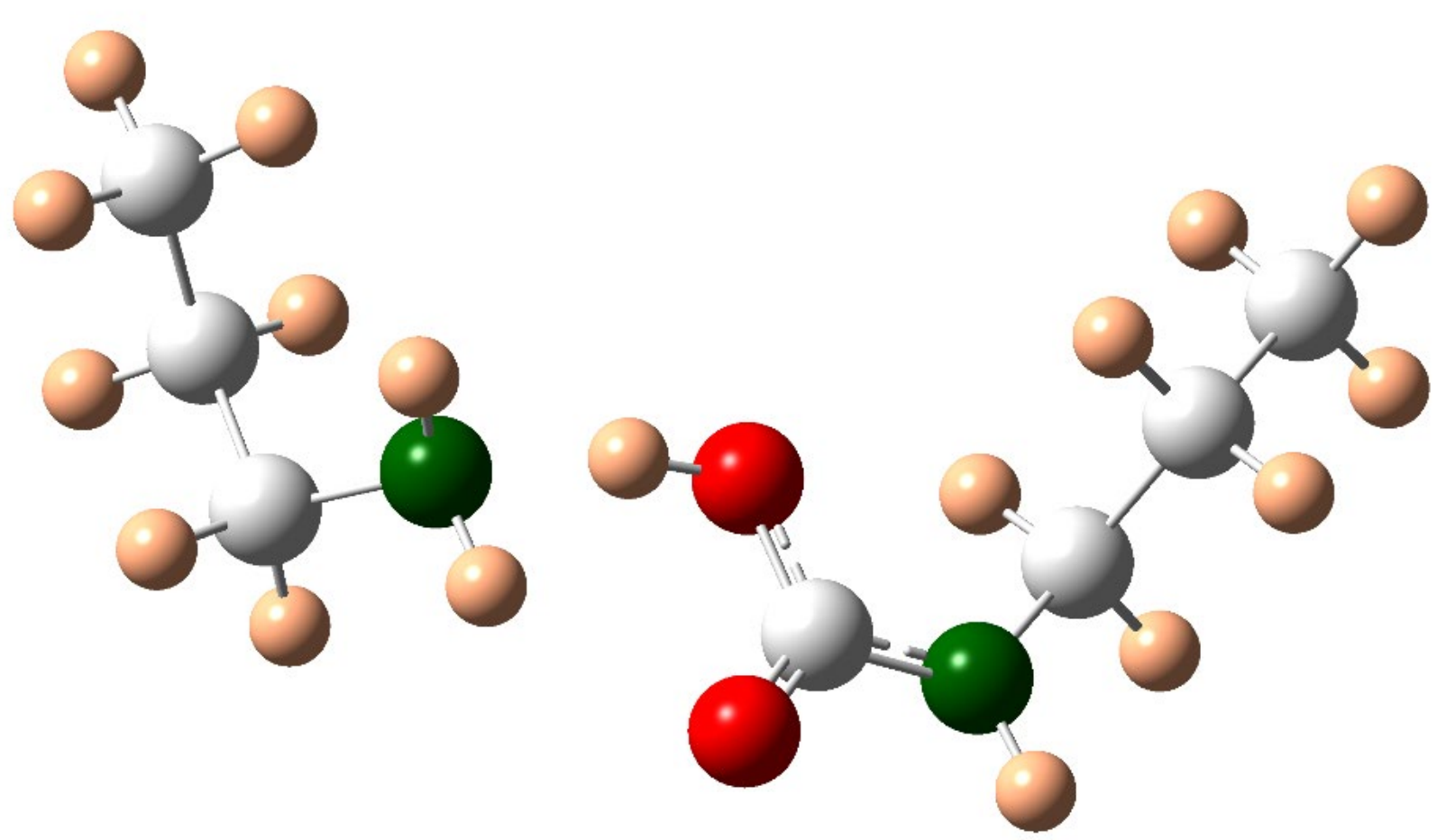

Figure S15. Optimized structure of carbamic acid species using Gaussian 16.[8]

4. References

(1) Cherevotan, A.; Raj, J.; Dheer, L.; Roy, S.; Sarkar, S.; Das, R.; Vinod, C. P.; Xu, S.; Wells, P.; Waghmare, U. V.; Peter, S. C. Operando Generated Ordered Heterogeneous Catalyst for the Selective Conversion of CO_2 to Methanol. *ACS Energy Lett.* **2021**, *6* (2), 509–516.

(2) Garvie, L. A. J.; Trif, L.; Cotto-Figueroa, D.; Asphaug, E.; Hoover, C. G. High Surface Area and Interconnected Nanoporosity of Clay-Rich Astromaterials. *Sci. Rep.* **2024**, *14* (1), 10358.

(3) Mathew, R.; Mazumder, A.; Kumar, P.; Matula, J.; Mohamed, S.; Brazda, P.; Hariharan, M.; Thomas, B. Unveiling the Topology of Partially Disordered Micro-Crystalline Nitro-Perylenediimide with X-Aggregate Stacking: An Integrated Approach. *Chem. Sci.* **2024**, *15* (2), 490–499.

(4) Mathew, R.; Thomas, B. Deciphering the Topology of Sitagliptin Using an Integrated Approach. *ACS Omega* **2025**, *10* (2), 2289–2295.

(5) Metz, G.; Wu, X.; Smith, S. O. Ramped-Amplitude Cross Polarization in Magic-Angle-Spinning NMR. *J. Magn. Reson. A* **1994**, *110* (2), 219–227.

(6) Kolodziejski, W.; Klinowski, J. Kinetics of Cross-Polarization in Solid-State NMR: A Guide for Chemists. *Chem. Rev.* **2002**, *102* (3), 613–628.

(7) Sakellariou, D.; Lesage, A.; Hodgkinson, P.; Emsley, L. Homonuclear Dipolar Decoupling in Solid-State NMR Using Continuous Phase Modulation. *Chem. Phys. Lett.* **2000**, *319* (3–4), 253–260.

(8) Frisch, M. J.; Trucks, G. W.; Schlegel, H. B.; Scuseria, G. E.; Robb, M. A.; Cheeseman, J. R.; Scalmani, G.; Barone, V.; Petersson, G. A.; Nakatsuji, H.; Li, X.; Caricato, M.; Marenich, A. V; Bloino, J.; Janesko, B. G.; Gomperts, R.; Mennucci, B.; Hratchian, H. P.; Ortiz, J. V; Izmaylov, A. F.; Sonnenberg, J. L.; Williams; Ding, F.; Lipparini, F.; Egidi, F.; Goings, J.; Peng, B.; Petrone, A.; Henderson, T.; Ranasinghe, D.; Zakrzewski, V. G.; Gao, J.; Rega, N.; Zheng, G.; Liang, W.; Hada, M.; Ehara, M.; Toyota, K.; Fukuda, R.; Hasegawa, J.; Ishida, M.; Nakajima, T.; Honda, Y.; Kitao, O.; Nakai, H.; Vreven, T.; Throssell, K.; Montgomery Jr., J. A.; Peralta, J. E.; Ogliaro, F.; Bearpark, M. J.; Heyd, J. J.; Brothers, E. N.; Kudin, K. N.; Staroverov, V. N.; Keith, T. A.; Kobayashi, R.; Normand, J.; Raghavachari, K.; Rendell, A. P.; Burant, J. C.; Iyengar, S. S.; Tomasi, J.; Cossi, M.; Millam, J. M.; Klene, M.; Adamo, C.; Cammi, R.; Ochterski, J. W.; Martin, R. L.; Morokuma, K.; Farkas, O.; Foresman, J. B.; Fox, D. J. Gaussian 16 Rev. C.01. Wallingford, CT 2016.
